# Trial data for precision analysis of a three-dimensional mandibular mechanical advantage

**DOI:** 10.1016/j.dib.2024.110402

**Published:** 2024-04-10

**Authors:** Dominique Ellen Carneiro, Giancarlo De La Torre Canales, Manuel Óscar Lagravère, Nara Hellen Campanha, Vanessa Migliorini Urban, Alfonso Sánchez-Ayala

**Affiliations:** a Department of Dentistry, University of Ponta Grossa, Ponta Grossa, Paraná, Brazil; b Division of Oral Diagnostics and Rehabilitation, Department of Dental Medicine, Karolinska Institutet, Huddinge, Stockholm, Sweden; c Egas Moniz Centre for Interdisciplinary Research (CiiEM), Egas Moniz School of Health & Science, Caparica, Almada, Portugal; d Department of Dentistry, Ingá University Center, Uningá, Maringá, Paraná, Brazil; e Orthodontic Graduate Program, University of Alberta, Edmonton, Alberta, Canada

**Keywords:** Biomechanical phenomena, Cone-beam computed tomography, Reliability, Muscles, Jaw, Planning

## Abstract

The data presented in this manuscript describe craniofacial landmark coordinate values, muscle and load moment arm lengths, and mechanical advantage rates for constructing a three-dimensional model of masticatory muscles. Cone-beam computed tomography scans from 30 subjects (aged 12–19 years, 16 females) were used. Thirty-six craniofacial landmarks were identified. Subsequently, the moment arms for 7 muscles and their corresponding load moment arms at incisor and molar positions were determined. Then, the three-dimensional mechanical advantage for each muscle and tooth position was calculated as the ratio of muscle moment arm to load moment arm. This procedure was repeated three times by a main examiner and once by two other examiners. The Friedman test and the square root of the 'method of moments' variance estimator were used to compare data among examiners and calculate random errors, respectively. Although the values for the craniofacial landmark coordinates and biomechanical variables are very close, differences were found between measurements, especially in the interexaminer comparisons. Values served as the basis for reliability (intraclass correlation coefficient) and errors (average mean of absolute differences) analysis in the research paper titled “A three-dimensional method to calculate mechanical advantage in mandibular function: Intra- and interexaminer reliability study,” published in the Journal of Orofacial Orthopedics.

Specifications Table

**Table:** 

Subject	Dentistry, Oral Surgery and Medicine
Specific subject area	Craniofacial research: Occlusion, Growth and Development, Orthodontics, Orthopaedics and Surgery
Type of data	Table Figure
How the data were acquired	In 30 craniofacial cone-beam computed tomographies of dentate subjects, 36 cephalometric landmarks, 3 skeletal reference planes, muscle and load force vectors, muscle and load moment arms, and mechanical advantage for 7 masticatory muscles (superficial masseter, anterior and posterior deep masseters, anterior and posterior temporals, and medial and lateral pterygoids) at molar and incisal bite positions were determined.
Data format	Millimetres (mm) in a three-dimensional space. Orientation was defined as the xz plane being coronal, the yz plane sagittal, and the xy plane axial. To establish landmarks, the coordinate system and the origin (0, 0, 0) provided by Avizo Fire software 8.1 (Mercury Computer Systems, Inc., Berlin, BE, Germany) were utilized. The biomechanical model was constructed using Cabri 3D software 2.1.2 (Cabrilog, Grenoble, Isère, France).
Description of data collection	Means and standard deviations of 5 trials corresponding to cephalometric landmarks' coordinates (x, y, and z), muscle and load moment arm lengths (mm), and mechanical advantage ratios for masticatory muscles on the right and left sides. The main examiner repeated all measurements three times (trials 1, 2, and 3). Two other examiners carried out these measures only once (in trials 4 and 5, respectively). Intraexaminer and interexaminer comparisons of the variables were conducted using the Friedman test (α = 0.05), with random error estimated by the square root of the 'method of moments' variance estimator (MME).
Data source location	Institution: University of Ponta Grossa City/Town/Region: Ponta Grossa/Paraná/Sul Country: Brazil Latitude and longitude for collected samples/data: GPS: 25°5′23″S 50°6′23″W
Data accessibility	The dataset has been deposited into an open repository and is available under the following permanent specifications: Repository name: Mendeley Data Data identification number: 10.17632/92m49gvgh6.1 Direct URL to data: https://doi.org/10.17632/92m49gvgh6.1
Related research article	A. Sánchez-Ayala, A. Sánchez-Ayala, R.C. Kolodzejezyk, V.M. Urban, M.O. Lagravère, N.H. Campanha, A three-dimensional method to calculate mechanical advantage in mandibular function: Intra- and interexaminer reliability study, J. Orofac. Orthop. 84 (2023) 321–339. https://doi.org/10.1007/s00056-022-00378–7.

## 1 Value of the Data

• The data serve as the numerical basis for the subsequent analysis of mandibular biomechanical reliability [1].
• The analysis can be used as a learning means for studying mandibular function [2] and applied during craniofacial diagnostics and planning for patients with orthodontic, orthopaedic, or surgical needs [3]. Moreover, the improvement of mandibular mechanical advantage can be verified after therapeutical changes in skeletal and dental relationships [4].
• Favourable moment arm designs can be simulated by considering the spatial movement of the teeth, occlusal plane, and muscular attachments [2], and can be addressed through the design of growth appliances or surgical guides.

## 2 Background

Mandibular movements are controlled by the neuromuscular system and are determined by the temporomandibular joints and dental occlusion. However, mandibular closing can be biomechanically conceived as a class III lever system. Under ideal conditions, the fulcrum is represented by the condyle, around which the mandible rotates as a rigid bar [5]. The effort or force is generated by the masticatory muscles, while the resistance or load is applied by tooth contact. Thus, the effort is placed between the fulcrum and resistance. A lever system will be in equilibrium when the product of the muscle force multiplied by the muscle moment arm is equal to the product of the bite force multiplied by the load moment arm. Muscle and load moment arms are defined as the distances between the fulcrum, and the muscle and the bite force vectors, respectively [4]. Therefore, a mechanical advantage, where the ratio of the muscle moment arm to the load moment arm is less than 1, would enhance the efficiency of the masticatory muscles in generating strength in a specific bite position for chewing food [6].

In Sánchez-Ayala et al. [1], both three-dimensional landmark coordinates and moment arms for mechanical advantage analysis were only indirectly reported. The study evaluated the intra- and interexaminer reliability of measurements using the Intraclass Correlation Coefficient (ICC) with a two-factor mixed model for absolute agreement. Intra- and interexaminer errors were calculated based on the average mean of absolute differences (AMAD), while intra- and interexaminer disagreements were estimated using the coefficient of variation (CV). However, the original values could not be included due to space limitations when presenting the material.

The present article provides the initial raw data for the aforementioned results: mean and standard deviation; intra- and interexaminer mean differences, evaluated using the Friedman test; and intra- and interexaminer random errors, calculated using the MME. Understanding these numbers is necessary because they illustrate the true extent of the differences. Small or large differences will be more or less relevant if the original data also corresponds. In this way, a given difference becomes smaller as the original values represent large magnitudes, and vice versa. Measurement values and their distributions reflect real data; through reliability tests, data is analysed to generalize the results to other potential scenarios. Thus, values with high reliability are not necessarily very similar.

## 3 Data Description

Means and standard deviations of trials 1, 2, and 3 corresponding to 36 cephalometric landmark coordinates (axes *x, y,* and *z*) in raw form are shown in Table 1 (intraexaminer comparison). Differences were found among trials for Gnathion (*y*), Orbitale (left: *x* and *y*), Coronoid (left: *z*), Maxillozygomatic (left: *z*), Pterygoid fovea (right: *y*), Pterygoid (right: *x*; left: *x* and *z*), and Zyd (right: *x*).

**Table 1:** Intraexaminer mean and standard deviation (SD) corresponding to landmarks' coordinates (mm).

Landmarks	Trial 1						Trial 2						Trial 3						Significance (p)		
	*x*		*y*		*z*		*x*		*y*		*z*		*x*		*y*		*z*		*x*	*y*	*z*
	Mean	SD	Mean	SD	Mean	SD	Mean	SD	Mean	SD	Mean	SD	Mean	SD	Mean	SD	Mean	SD			
ELSA	−19.70	4.65	11.08	9.20	60.71	8.01	−19.65	4.61	11.11	9.20	60.79	8.00	−19.62	4.78	11.17	9.38	60.77	7.86	0.417	0.712	0.342
Nasion	−20.82	4.16	−60.06	8.31	92.06	6.23	−20.72	4.21	−60.11	8.30	92.17	6.24	−20.76	4.12	−60.16	8.33	92.02	6.11	0.195	0.515	0.407
Gnathion	−20.75	5.22	−60.75	11.63	−19.53	5.66	−20.64	5.22	−60.64	11.83	−19.63	5.71	−20.54	5.24	−60.31	11.71	−19.84	5.62	0.072	**0.002**	0.072
Incisal	−20.67	4.84	64.76	10.81	17.80	6.01	−20.70	4.84	64.72	10.53	17.64	6.20	−20.60	4.81	64.71	10.51	17.70	6.22	0.327	0.356	0.792

*Right side*																					

Foramen spinosum	−50.41	5.32	11.70	9.13	60.74	8.01	−50.41	5.41	11.63	9.05	60.84	8.09	−50.27	5.83	11.76	9.35	60.93	7.97	0.697	0.291	0.721
Orbitale	−55.85	5.20	−47.28	8.48	64.72	6.96	−55.57	5.29	−47.53	8.45	64.67	6.97	−55.70	5.37	−47.41	8.41	64.67	6.98	0.239	0.067	0.851
Porion	−73.27	6.32	28.44	10.13	63.40	9.78	−73.13	5.94	28.41	10.14	63.32	9.80	−73.27	6.38	28.29	10.03	63.39	9.68	0.356	0.905	0.497
Condylion	−68.43	6.41	15.55	9.75	62.63	8.98	−68.39	6.43	15.54	9.67	62.74	8.87	−68.30	6.50	15.60	9.78	62.75	8.84	0.648	0.122	0.085
Gonion	−64.80	7.02	7.70	10.80	7.33	6.93	−64.76	7.06	8.33	10.56	8.00	6.46	−64.91	6.91	7.87	10.75	7.49	6.56	0.506	0.273	0.088
Coronoid	−66.30	5.52	−16.32	9.56	57.68	7.77	−66.33	5.47	−16.35	9.55	57.71	7.82	−66.36	5.54	−16.34	9.55	57.75	7.73	0.942	0.943	0.587
Ramus	−59.41	5.35	−18.48	9.85	24.06	5.59	−59.46	5.37	−18.48	9.83	24.23	5.78	−59.49	5.39	−18.50	9.81	24.13	5.91	0.792	0.741	0.905
Maxillozygomatic	−62.75	4.81	−35.69	9.11	46.61	6.42	−62.93	5.07	−35.62	9.11	46.50	6.48	−62.66	5.85	−35.88	9.08	46.47	6.52	0.435	0.905	0.218
Temporozygomatic	−78.58	6.09	−11.04	9.77	57.97	7.67	−78.50	6.00	−11.09	9.73	57.79	7.60	−78.67	6.04	−11.03	9.66	57.85	7.63	0.118	0.741	0.122
Pterygoid fovea	−67.73	6.34	10.79	9.36	52.13	8.19	−67.80	6.41	10.77	9.68	52.19	8.31	−67.66	6.31	10.51	9.55	51.90	8.42	0.295	**0.020**	0.072
Pterygoid	−44.55	5.62	−6.75	9.98	43.75	6.87	−45.13	5.77	−5.97	10.14	43.36	6.91	−45.06	5.62	−6.03	10.15	43.50	7.27	**0.014**	0.356	0.497
Zyd	−78.77	6.42	6.20	9.36	57.56	8.53	−78.82	6.54	6.30	9.22	57.58	8.50	−79.09	6.43	6.18	9.38	57.65	8.51	**0.010**	0.515	0.648
Molar	1.03	4.26	−37.37	10.50	16.86	5.68	1.00	4.15	−37.57	10.59	16.70	5.76	1.04	4.30	−37.47	10.63	16.73	5.72	0.792	0.875	0.889
Maa	−61.20	5.76	−9.75	10.04	18.49	5.79	−61.22	5.79	−9.54	9.93	18.82	5.78	−61.30	5.76	−9.73	9.99	18.58	5.93	0.374	0.577	0.374
Map	−63.00	6.32	−1.06	10.37	12.95	6.25	−63.00	6.37	−0.65	10.18	13.44	6.01	−63.10	6.27	−0.97	10.30	13.07	6.16	0.337	0.662	0.485
Zys	−73.27	5.38	−19.31	9.41	54.17	7.11	−73.28	5.41	−19.32	9.40	54.01	7.11	−73.30	5.77	−19.37	9.36	54.05	7.15	0.498	0.991	0.198
*Left side*																					

Foramen spinosum	11.20	4.68	10.45	9.46	60.70	8.20	11.28	4.60	10.56	9.54	60.72	8.13	11.15	4.54	10.55	9.60	60.60	8.06	0.195	0.648	0.832
Orbitale	14.09	3.45	−48.85	8.41	64.39	7.38	14.59	3.88	−48.40	8.96	64.47	7.34	14.55	3.91	−48.61	8.88	64.44	7.35	**0.048**	**0.045**	0.697
Porion	35.42	4.89	26.07	10.60	62.63	8.94	35.46	5.19	26.09	10.54	62.57	9.02	35.54	5.04	26.03	10.67	62.56	8.92	0.301	0.531	0.587
Condylion	29.66	4.64	14.02	10.01	62.50	8.43	29.70	4.72	13.95	10.05	62.47	8.43	29.59	4.74	14.04	10.02	62.50	8.44	0.587	0.056	0.744
Gonion	26.29	4.73	6.89	11.25	8.42	7.34	26.20	4.77	7.40	11.53	9.00	7.14	26.17	4.73	7.08	11.36	8.59	6.90	0.587	0.072	0.239
Coronoid	26.58	4.71	−17.54	9.57	57.35	7.56	26.65	4.70	−17.55	9.60	57.38	7.56	26.70	4.76	−17.50	9.64	57.46	7.56	0.792	0.905	**0.018**
Ramus	19.25	4.20	−19.77	10.48	23.58	5.87	19.36	4.41	−19.67	10.31	23.91	5.14	19.31	4.20	−19.82	10.34	23.48	5.79	0.497	0.131	0.177
Maxillozygomatic	22.31	5.70	−37.18	9.53	46.60	7.00	22.45	5.46	−37.33	9.36	46.32	7.06	22.01	5.29	−37.31	9.31	46.33	7.02	0.239	0.569	**0.026**
Temporozygomatic	38.44	4.48	−13.94	10.25	57.46	7.98	38.67	4.76	−13.38	10.28	57.56	7.97	38.52	4.69	−13.55	10.20	57.42	8.08	0.400	0.239	0.400
Pterygoid fovea	28.64	4.59	9.27	9.93	52.27	8.09	28.75	4.46	9.03	9.85	52.19	8.26	28.60	4.66	9.04	9.80	51.95	8.14	0.497	0.131	0.435
Pterygoid	4.77	4.34	−7.32	9.87	43.72	6.26	5.02	4.10	−7.40	10.00	43.18	6.17	5.22	4.09	−7.40	9.91	42.91	6.11	**0.016**	0.497	**0.026**
Zyd	38.90	4.68	4.36	9.94	57.58	8.29	39.16	4.69	4.38	9.78	57.62	8.22	39.22	4.59	4.35	9.91	57.62	8.19	0.079	0.291	0.721
Molar	−41.71	5.46	−37.05	9.88	17.33	5.47	−41.68	5.46	−36.99	9.94	17.22	5.46	−41.68	5.50	−36.94	10.03	17.22	5.52	0.741	0.531	0.758
Maa	21.62	4.21	−10.89	10.62	18.52	6.20	21.64	4.37	−10.66	10.59	18.94	5.59	21.59	4.22	−10.85	10.59	18.51	5.97	0.892	0.204	0.670
Map	23.94	4.40	−2.06	10.87	13.50	6.70	23.90	4.49	−1.67	11.02	14.01	6.28	23.88	4.41	−1.93	10.94	13.59	6.36	0.704	0.404	0.311
Zys	33.12	4.74	−21.62	9.87	53.87	7.55	33.30	4.84	−21.31	9.85	53.85	7.56	33.04	4.71	−21.44	9.79	53.75	7.63	0.723	0.568	0.712

*p* < 0.05 are shown in bold type.

Table 2 describes interexaminer comparisons (trials 1, 4, and 5) for 36 raw cephalometric landmark coordinates (axes *x, y,* and *z*). ELSA (*x*), Gnathion (*x, y,* and *z*), Incisal (*x* and *z*), Foramen spinosum (right: *x* and *y;* left*: x*), Orbitale (right and left: *x, y*, and *z*), Porion (left: *x*), Condylion (right: *x* and *y*; left: *x, y*, and *z*), Gonion (right and left: *x, y*, and *z*), Coronoid (right: *x* and *y*; left: *x, y*, and *z*), Ramus (right and left: *x, y*, and *z*), Maxillozygomatic (right and left: *y* and *z*), Temporozygomatic (right: *x*; left: *x* and *z*), Pterygoid fovea (right and left: *x, y*, and *z*), Pterygoid (right: *x,y*, and *z*; left: *x* and *z*), Zyd (right: *y*), Molar (left: *y* and *z*), Maa (right and left: *x, y*, and *z*), Map (right: *y* and *z*; left: *x, y*, and *z*), and Zys (right: *x, y*, and *z*; left: *x* and *y*) presented differences.

**Table 2:** Interexaminer mean and standard deviation (SD) corresponding to landmarks' coordinates (mm).

Landmarks	Trial 1						Trial 4						Trial 5						Significance (p)		
	*x*		*y*		*z*		*x*		*y*		*z*		*x*		*y*		*z*		*x*	*y*	*z*
	Mean	SD	Mean	SD	Mean	SD	Mean	SD	Mean	SD	Mean	SD	Mean	SD	Mean	SD	Mean	SD			
ELSA	−19.70	4.65	11.08	9.20	60.71	8.01	−19.68	4.54	10.70	9.09	60.73	8.20	−19.47	4.47	10.62	9.37	60.40	7.88	**0.024**	0.075	0.154
Nasion	−20.82	4.16	−60.06	8.31	92.06	6.23	−20.69	4.14	−60.16	8.39	91.84	6.19	−20.75	4.20	−60.07	8.26	92.08	6.29	0.131	0.356	0.587
Gnathion	−20.75	5.22	−60.75	11.63	−19.53	5.66	−20.62	5.11	−61.63	11.28	−18.34	5.64	−21.45	5.08	−60.70	11.82	−19.47	5.61	**0.000**	**0.000**	**0.000**
Incisal	−20.67	4.84	64.76	10.81	17.80	6.01	−20.90	4.81	65.03	10.64	17.42	6.11	−20.63	4.63	65.22	10.59	17.48	6.21	**0.003**	0.061	**0.012**

*Right side*																					

Foramen spinosum	−50.41	5.32	11.70	9.13	60.74	8.01	−50.66	5.73	11.15	8.94	60.67	8.29	−50.27	5.59	11.35	9.25	60.20	8.01	**0.039**	**0.006**	0.072
Orbitale	−55.85	5.20	−47.28	8.48	64.72	6.96	−62.46	5.67	−42.79	8.31	67.19	7.43	−57.42	5.71	−46.97	8.21	64.59	6.97	**0.000**	**0.000**	**0.000**
Porion	−73.27	6.32	28.44	10.13	63.40	9.78	−73.43	5.65	28.33	10.06	63.38	10.04	−72.85	6.32	28.18	10.23	63.23	9.82	0.202	0.648	0.088
Condylion	−68.43	6.41	15.55	9.75	62.63	8.98	−69.10	6.75	15.81	9.70	62.85	8.79	−69.36	5.98	15.31	9.46	62.99	9.07	**0.007**	**0.001**	0.179
Gonion	−64.80	7.02	7.70	10.80	7.33	6.93	−65.27	6.98	6.58	10.94	6.05	6.70	−65.85	6.79	5.52	10.90	5.72	6.89	**0.000**	**0.000**	**0.001**
Coronoid	−66.30	5.52	−16.32	9.56	57.68	7.77	−66.62	5.69	−16.11	9.35	57.51	7.72	−65.92	5.51	−16.70	9.48	57.55	7.75	**0.000**	**0.012**	0.118
Ramus	−59.41	5.35	−18.48	9.85	24.06	5.59	−59.09	5.30	−19.42	9.93	22.10	5.78	−58.12	5.50	−21.66	10.11	18.42	5.69	**0.000**	**0.000**	**0.000**
Maxillozygomatic	−62.75	4.81	−35.69	9.11	46.61	6.42	−62.55	5.08	−36.00	9.17	46.33	6.56	−62.72	4.94	−36.77	9.22	46.19	6.44	0.239	**0.000**	**0.045**
Temporozygomatic	−78.58	6.09	−11.04	9.77	57.97	7.67	−78.40	6.11	−11.22	9.81	57.74	7.61	−79.23	6.08	−11.30	10.04	57.71	7.59	**0.000**	0.273	0.061
Pterygoid fovea	−67.73	6.34	10.79	9.36	52.13	8.19	−66.79	6.21	9.99	9.16	49.80	7.69	−67.94	6.61	11.34	9.54	53.34	8.19	**0.000**	**0.000**	**0.000**
Pterygoid	−44.55	5.62	−6.75	9.98	43.75	6.87	−44.71	5.48	−5.53	10.18	44.53	7.75	−43.93	5.61	−6.98	9.40	44.97	7.30	**0.007**	**0.002**	**0.048**
Zyd	−78.77	6.42	6.20	9.36	57.56	8.53	−78.86	6.23	5.65	9.16	57.51	8.41	−79.02	6.53	5.80	9.33	57.58	8.57	0.273	**0.000**	0.792
Molar	1.03	4.26	−37.37	10.50	16.86	5.68	0.99	4.10	−37.43	10.41	17.18	5.70	0.90	4.58	−37.30	10.25	17.38	5.77	0.531	0.905	0.239
Maa	−61.20	5.76	−9.75	10.04	18.49	5.79	−61.15	5.72	−10.75	10.13	16.73	5.91	−60.70	5.76	−12.60	10.17	14.19	5.73	**0.001**	**0.000**	**0.000**
Map	−63.00	6.32	−1.06	10.37	12.95	6.25	−63.21	6.27	−2.12	10.48	11.43	6.22	−63.28	6.19	−3.59	10.44	9.96	6.15	0.056	**0.000**	**0.000**
Zys	−73.27	5.38	−19.31	9.41	54.17	7.11	−73.12	5.53	−19.50	9.49	53.93	7.15	−73.71	5.43	−19.81	9.68	53.85	7.10	**0.000**	**0.000**	**0.003**
*Left side*																					

Foramen spinosum	11.20	4.68	10.45	9.46	60.70	8.20	11.45	4.29	10.26	9.49	60.78	8.38	11.53	4.24	9.86	9.95	60.60	8.04	**0.010**	0.202	0.670
Orbitale	14.09	3.45	−48.85	8.41	64.39	7.38	21.98	4.09	−43.81	8.92	67.24	7.73	14.95	4.32	−48.70	8.92	64.36	7.29	**0.000**	**0.000**	**0.000**
Porion	35.42	4.89	26.07	10.60	62.63	8.94	35.06	5.22	26.58	10.25	62.83	9.26	35.56	5.81	25.65	10.63	62.57	9.13	**0.036**	0.061	0.648
Condylion	29.66	4.64	14.02	10.01	62.50	8.43	29.39	5.00	14.29	10.16	62.65	8.38	30.57	4.84	13.75	9.76	62.46	8.51	**0.002**	**0.032**	**0.008**
Gonion	26.29	4.73	6.89	11.25	8.42	7.34	26.78	4.85	4.71	11.24	5.67	6.73	27.15	4.79	4.74	11.28	6.46	6.46	**0.000**	**0.000**	**0.000**
Coronoid	26.58	4.71	−17.54	9.57	57.35	7.56	26.93	4.57	−17.37	9.45	57.17	7.62	26.47	4.50	−17.69	9.38	57.29	7.58	**0.000**	**0.003**	**0.015**
Ramus	19.25	4.20	−19.77	10.48	23.58	5.87	19.22	4.30	−20.13	10.25	22.60	5.87	17.04	4.48	−23.31	10.60	17.30	5.98	**0.000**	**0.000**	**0.000**
Maxillozygomatic	22.31	5.70	−37.18	9.53	46.60	7.00	22.02	5.46	−37.33	9.31	46.19	6.98	21.51	5.65	−38.25	9.44	45.87	7.28	0.741	**0.000**	**0.000**
Temporozygomatic	38.44	4.48	−13.94	10.25	57.46	7.98	38.29	4.45	−13.55	10.20	57.48	8.17	39.17	4.91	−13.25	10.60	57.74	7.99	**0.000**	0.195	**0.012**
Pterygoid fovea	28.64	4.59	9.27	9.93	52.27	8.09	27.64	4.43	7.48	9.98	49.56	7.77	28.50	4.41	10.14	9.95	53.29	8.20	**0.000**	**0.000**	**0.000**
Pterygoid	4.77	4.34	−7.32	9.87	43.72	6.26	3.58	4.19	−7.67	9.90	45.50	7.66	4.03	4.28	−7.37	9.45	44.63	7.26	**0.000**	0.792	**0.025**
Zyd	38.90	4.68	4.36	9.94	57.58	8.29	38.79	4.54	4.12	9.68	57.48	8.24	38.96	4.54	4.04	9.65	57.57	8.36	0.967	0.356	0.792
Molar	−41.71	5.46	−37.05	9.88	17.33	5.47	−41.71	5.34	−36.51	10.14	17.51	5.37	−41.59	5.67	−36.42	10.28	17.77	5.34	0.875	**0.000**	**0.002**
Maa	21.62	4.21	−10.89	10.62	18.52	6.20	21.74	4.33	−11.85	10.47	16.95	5.98	20.40	4.41	−13.96	10.67	13.69	5.88	**0.000**	**0.000**	**0.000**
Map	23.94	4.40	−2.06	10.87	13.50	6.70	24.23	4.52	−3.63	10.79	11.34	6.28	23.76	4.52	−4.66	10.91	10.09	6.04	**0.000**	**0.000**	**0.000**
Zys	33.12	4.74	−21.62	9.87	53.87	7.55	32.90	4.64	−21.43	9.78	53.74	7.68	33.34	4.83	−21.51	10.10	53.83	7.62	**0.000**	**0.001**	0.641

*p* < 0.05 are shown in bold type.

Muscle and load moment arms lengths and mechanical advantage ratios (mean and standard deviation) of 7 masticatory muscles are described for intraexaminer and interexaminer comparisons in Table 3. There were intraexaminer differences among trials to muscle moment arm (left: superficial masseter) and mechanical advantage (right: medial pterygoid; left: superficial masseter and anterior deep masseter). For interexaminer comparisons, muscle moment arm (right and left: superficial masseter, posterior temporal, and lateral pterygoid; left: posterior deep masseter), load moment arm (right and left: molar; right: incisive), mechanical advantage to molar position (right and left: superficial masseter, anterior deep masseter, and lateral pterygoid; right: anterior temporal; left: posterior deep masseter), and mechanical advantage to incisal position (right and left: superficial masseter, posterior temporal, and lateral pterygoid; left: posterior deep masseter) showed differences.

**Table 3:** Mean and standard deviation (SD) corresponding to biomechanics variables (mm).

Biomechanics variables		Trial 1		Trial 2		Trial 3		Trial 4		Trial 5		Significance (p)	
		Mean	SD	Mean	SD	Mean	SD	Mean	SD	Mean	SD	Intraexaminer	Interexaminer
*Right side*													

MUSCLE MOMENT ARM	Superficial masseter	34.60	3.31	34.57	3.33	34.77	3.19	35.53	3.29	35.43	3.56	0.426	**0.000**
	Anterior deep masseter	32.60	3.35	32.57	3.44	32.70	3.23	32.73	3.03	32.83	3.16	0.416	0.854
	Posterior deep masseter	14.43	3.08	14.37	3.35	14.67	3.13	14.90	2.06	14.33	2.99	0.119	0.475
	Anterior temporal	32.47	3.31	32.40	3.43	32.60	3.23	32.40	3.04	32.83	3.21	0.199	0.124
	Posterior temporal	28.83	2.91	28.80	2.89	28.87	2.96	28.17	2.93	28.70	3.05	0.794	**0.001**
	Medial pterygoid	37.73	2.39	37.17	2.93	37.10	2.93	37.33	2.90	38.20	2.61	0.055	0.170
	Lateral pterygoid	10.13	1.81	10.00	1.64	10.33	1.71	12.53	2.37	9.00	2.10	0.186	**0.000**

LOAD MOMENT ARM	Molar	71.17	5.44	71.17	5.34	71.20	5.37	70.23	5.23	70.47	5.83	0.942	**0.010**
	Incisal	103.13	5.42	103.27	5.07	103.33	5.27	103.53	5.38	103.83	5.23	0.575	**0.034**

MECHANICAL ADVANTAGE	Molar position												
	Superficial masseter	0.49	0.04	0.49	0.05	0.49	0.05	0.51	0.05	0.50	0.05	0.585	**0.000**
	Anterior deep masseter	0.46	0.04	0.46	0.05	0.46	0.04	0.47	0.04	0.47	0.04	0.766	**0.001**
	Posterior deep masseter	0.20	0.05	0.20	0.05	0.21	0.05	0.21	0.04	0.21	0.05	0.262	0.552
	Anterior temporal	0.46	0.04	0.46	0.04	0.46	0.04	0.46	0.04	0.47	0.04	0.616	**0.022**
	Posterior temporal	0.41	0.04	0.41	0.04	0.41	0.04	0.40	0.04	0.41	0.04	0.888	0.056
	Medial pterygoid	0.53	0.03	0.52	0.04	0.52	0.04	0.53	0.04	0.55	0.04	0.111	0.296
	Lateral pterygoid	0.14	0.03	0.14	0.03	0.15	0.03	0.18	0.03	0.13	0.03	0.141	**0.000**

	Incisal position												
	Superficial masseter	0.34	0.03	0.33	0.03	0.34	0.03	0.34	0.03	0.34	0.03	0.326	**0.002**
	Anterior deep masseter	0.32	0.03	0.32	0.03	0.32	0.02	0.32	0.03	0.32	0.03	0.589	0.868
	Posterior deep masseter	0.14	0.03	0.14	0.03	0.14	0.03	0.15	0.02	0.14	0.03	0.220	0.337
	Anterior temporal	0.31	0.03	0.31	0.03	0.32	0.03	0.31	0.03	0.32	0.03	0.238	0.358
	Posterior temporal	0.28	0.03	0.28	0.02	0.28	0.02	0.27	0.02	0.28	0.02	0.931	**0.003**
	Medial pterygoid	0.37	0.02	0.36	0.03	0.36	0.02	0.36	0.02	0.37	0.02	**0.031**	0.180
	Lateral pterygoid	0.10	0.02	0.10	0.02	0.10	0.02	0.12	0.02	0.09	0.02	0.254	**0.000**
*Left side*													

MUSCLE MOMENT ARM	Superficial masseter	35.00	2.80	34.47	2.70	34.87	2.76	35.83	2.67	35.17	3.29	**0.026**	**0.001**
	Anterior deep masseter	32.43	3.23	32.20	3.10	32.33	3.33	32.40	3.17	32.20	3.41	0.128	0.645
	Posterior deep masseter	14.03	2.34	14.23	2.46	14.47	2.33	14.53	2.21	13.43	2.57	0.193	**0.008**
	Anterior temporal	32.23	2.97	32.13	3.04	32.17	3.07	32.03	2.93	32.17	3.28	0.751	0.298
	Posterior temporal	28.33	2.60	28.27	2.64	28.33	2.55	27.80	2.92	28.17	3.25	0.766	**0.018**
	Medial pterygoid	37.63	2.83	37.73	2.88	37.90	2.77	37.90	2.77	38.30	2.93	0.267	0.482
	Lateral pterygoid	9.57	1.85	9.53	1.81	9.87	1.68	13.47	1.98	8.43	2.39	0.108	**0.000**

LOAD MOMENT ARM	Molar	71.03	4.53	71.37	4.39	71.37	4.51	70.10	4.34	70.70	4.53	0.158	**0.011**
	Incisal	103.03	4.66	103.10	4.42	103.13	4.55	103.23	4.52	103.63	4.52	0.814	0.364

MECHANICAL ADVANTAGE	Molar position												
	Superficial masseter	0.49	0.04	0.48	0.04	0.49	0.04	0.51	0.04	0.50	0.05	**0.008**	**0.000**
	Anterior deep masseter	0.46	0.04	0.45	0.04	0.45	0.04	0.46	0.04	0.46	0.04	**0.017**	**0.046**
	Posterior deep masseter	0.20	0.04	0.20	0.04	0.20	0.04	0.21	0.04	0.19	0.04	0.408	**0.003**
	Anterior temporal	0.45	0.04	0.45	0.04	0.45	0.04	0.46	0.04	0.46	0.04	0.128	0.673
	Posterior temporal	0.40	0.04	0.40	0.03	0.40	0.03	0.40	0.04	0.40	0.05	0.358	0.523
	Medial pterygoid	0.53	0.03	0.53	0.04	0.53	0.03	0.54	0.04	0.54	0.04	0.957	0.075
	Lateral pterygoid	0.14	0.03	0.13	0.03	0.14	0.03	0.19	0.03	0.12	0.04	0.317	**0.000**
	Incisal position												
	Superficial masseter	0.34	0.03	0.33	0.03	0.34	0.03	0.35	0.03	0.34	0.03	0.085	**0.001**
	Anterior deep masseter	0.32	0.03	0.31	0.02	0.31	0.03	0.31	0.03	0.31	0.03	0.225	0.065
	Posterior deep masseter	0.14	0.03	0.14	0.02	0.14	0.02	0.14	0.02	0.13	0.03	0.343	**0.021**
	Anterior temporal	0.31	0.02	0.31	0.02	0.31	0.02	0.31	0.02	0.31	0.03	0.595	0.170
	Posterior temporal	0.28	0.02	0.27	0.02	0.27	0.02	0.27	0.02	0.27	0.03	0.673	**0.006**
	Medial pterygoid	0.37	0.02	0.37	0.02	0.37	0.02	0.37	0.02	0.37	0.02	0.486	0.757
	Lateral pterygoid	0.09	0.02	0.09	0.02	0.10	0.02	0.13	0.02	0.08	0.02	0.126	**0.000**

*p* < 0.05 are shown in bold type.

Results only indicated an interexaminer error of 1.55 mm for the method of moments variance estimator on the right Porion *x*-axis (Table 4, Table 5). Errors lower than 1.5 mm are considered clinically acceptable [5].

**Table 4:** Intra- and interexaminer errors of landmarks coordinates (mm).

Landmarks	Intraexaminer									Interexaminer								
	MME			Lower limit			Upper limit			MME			Lower limit			Upper limit		
	*x*	*y*	*z*	*x*	*y*	*z*	*x*	*y*	*z*	*x*	*y*	*z*	*x*	*y*	*z*	*x*	*y*	*z*
ELSA	0.11	0.16	0.37	0.09	0.13	0.30	0.12	0.17	0.40	0.19	0.78	0.39	0.15	0.62	0.31	0.20	0.84	0.42
Nasion	0.10	0.10	0.35	0.08	0.08	0.28	0.13	0.14	0.47	0.12	0.16	0.51	0.10	0.13	0.41	0.16	0.22	0.69
Gnathion	0.16	0.24	0.23	0.13	0.19	0.18	0.21	0.32	0.31	0.28	0.44	0.39	0.23	0.35	0.31	0.38	0.59	0.53
Incisal	0.16	0.30	0.45	0.13	0.24	0.36	0.21	0.41	0.61	0.51	0.31	0.46	0.41	0.24	0.36	0.69	0.41	0.61

*Right side*																		

Foramen spinosum	0.35	0.27	0.69	0.28	0.22	0.55	0.46	0.37	0.93	0.31	0.35	0.54	0.24	0.28	0.43	0.41	0.47	0.73
Orbitale	0.31	0.24	0.07	0.25	0.19	0.05	0.42	0.32	0.09	1.14	0.89	0.66	0.91	0.71	0.53	1.53	1.20	0.89
Porion	1.13	0.32	0.24	0.90	0.26	0.19	1.52	0.43	0.32	**1.55**	0.59	0.31	1.23	0.47	0.25	2.08	0.79	0.42
Condylion	0.18	0.07	0.23	0.14	0.06	0.18	0.24	0.10	0.31	0.79	0.30	0.43	0.63	0.24	0.34	1.06	0.40	0.58
Gonion	0.26	0.43	0.52	0.21	0.34	0.41	0.35	0.58	0.70	0.32	0.95	0.71	0.26	0.75	0.56	0.43	1.27	0.95
Coronoid	0.17	0.13	0.15	0.14	0.11	0.12	0.23	0.18	0.21	0.34	0.32	0.18	0.27	0.25	0.14	0.46	0.43	0.24
Ramus	0.20	0.25	0.45	0.16	0.20	0.36	0.27	0.34	0.60	0.42	0.74	0.98	0.34	0.59	0.78	0.57	0.99	1.32
Maxillozygomatic	0.95	0.39	0.33	0.75	0.31	0.26	1.27	0.52	0.44	0.54	0.41	0.31	0.43	0.32	0.25	0.73	0.55	0.42
Temporozygomatic	0.14	0.16	0.15	0.11	0.13	0.12	0.18	0.21	0.21	0.26	0.32	0.21	0.21	0.25	0.16	0.36	0.42	0.28
Pterygoid fovea	0.16	0.31	0.34	0.13	0.25	0.27	0.22	0.42	0.46	0.43	0.46	0.96	0.34	0.37	0.76	0.58	0.62	1.29
Pterygoid	0.30	0.55	0.47	0.24	0.44	0.38	0.40	0.74	0.64	0.37	1.02	0.65	0.30	0.82	0.52	0.50	1.38	0.87
Zyd	0.22	0.36	0.09	0.17	0.29	0.07	0.29	0.48	0.12	0.38	0.49	0.16	0.30	0.39	0.13	0.51	0.66	0.22
Molar	0.14	0.12	0.19	0.11	0.09	0.15	0.19	0.16	0.25	0.56	0.54	0.29	0.44	0.43	0.23	0.75	0.73	0.39
Maa	0.11	0.24	0.45	0.09	0.19	0.36	0.12	0.26	0.48	0.28	0.61	0.74	0.22	0.49	0.59	0.29	0.66	0.80
Map	0.18	0.37	0.49	0.14	0.29	0.39	0.19	0.39	0.53	0.20	0.73	0.70	0.16	0.58	0.56	0.21	0.79	0.75
Zys	0.25	0.44	0.91	0.20	0.35	0.73	0.27	0.47	0.98	0.26	0.29	0.20	0.21	0.23	0.16	0.28	0.31	0.21
*Left side*																		

Foramen spinosum	0.18	0.16	0.31	0.14	0.13	0.25	0.24	0.22	0.42	0.49	1.33	0.48	0.39	1.06	0.38	0.66	1.79	0.65
Orbitale	0.40	0.45	0.09	0.32	0.36	0.07	0.54	0.60	0.12	1.23	0.67	0.59	0.98	0.53	0.47	1.65	0.90	0.80
Porion	0.80	0.36	0.30	0.64	0.28	0.24	1.08	0.48	0.40	1.25	0.68	0.47	1.00	0.54	0.38	1.69	0.91	0.63
Condylion	0.13	0.08	0.08	0.10	0.06	0.06	0.18	0.11	0.10	0.72	0.32	0.33	0.57	0.26	0.26	0.97	0.43	0.44
Gonion	0.29	0.51	0.62	0.23	0.41	0.50	0.39	0.69	0.84	0.29	0.69	0.87	0.23	0.55	0.70	0.39	0.92	1.17
Coronoid	0.12	0.14	0.09	0.10	0.11	0.07	0.16	0.19	0.12	0.21	0.32	0.18	0.17	0.25	0.15	0.28	0.43	0.25
Ramus	0.17	0.24	0.55	0.13	0.19	0.44	0.22	0.33	0.74	0.60	0.82	1.09	0.47	0.65	0.87	0.80	1.10	1.46
Maxillozygomatic	0.99	0.27	0.24	0.78	0.21	0.19	1.32	0.36	0.33	1.44	0.40	0.60	1.15	0.32	0.48	1.94	0.54	0.81
Temporozygomatic	0.35	0.96	0.22	0.28	0.76	0.17	0.47	1.29	0.29	0.57	1.40	0.29	0.45	1.12	0.23	0.77	1.89	0.40
Pterygoid fovea	0.23	0.22	0.31	0.18	0.17	0.25	0.31	0.30	0.42	0.48	0.57	0.95	0.38	0.46	0.76	0.64	0.77	1.28
Pterygoid	0.27	0.23	0.40	0.21	0.19	0.32	0.36	0.31	0.54	0.43	0.77	0.79	0.34	0.62	0.63	0.58	1.04	1.06
Zyd	0.28	0.23	0.11	0.22	0.19	0.09	0.37	0.31	0.15	0.37	0.27	0.17	0.30	0.22	0.13	0.50	0.36	0.23
Molar	0.29	0.26	0.35	0.23	0.21	0.28	0.39	0.35	0.46	0.69	0.39	0.36	0.55	0.31	0.28	0.93	0.52	0.48
Maa	0.12	0.23	0.40	0.09	0.19	0.32	0.13	0.25	0.43	0.41	0.65	0.89	0.33	0.51	0.71	0.44	0.69	0.95
Map	0.17	0.31	0.41	0.13	0.25	0.33	0.18	0.33	0.44	0.23	0.59	0.83	0.18	0.47	0.66	0.24	0.63	0.89
Zys	0.30	0.44	0.75	0.24	0.35	0.60	0.32	0.48	0.80	0.46	0.92	0.24	0.36	0.73	0.19	0.49	0.98	0.26

MME: Method of moments variance estimator.

Average values >1.5 mm are shown in bold type.

**Table 5:** Intra- and Interexaminer errors to biomechanics variables.

Biomechanics variables	Intraexaminer			Interexaminer		
	MME	Lower limit	Upper limit	MME	Lower limit	Upper limit
*Right side*						
MUSCLE MOMENT ARM (mm)						

Superficial masseter	0.22	0.17	0.29	0.41	0.33	0.55
Anterior deep masseter	0.21	0.17	0.29	0.35	0.28	0.48
Posterior deep masseter	0.23	0.18	0.31	0.81	0.65	1.09
Anterior temporal	0.21	0.17	0.29	0.33	0.27	0.45
Posterior temporal	0.19	0.15	0.25	0.35	0.28	0.47
Medial pterygoid	0.42	0.34	0.57	0.75	0.60	1.01
Lateral pterygoid	0.33	0.26	0.44	0.82	0.66	1.11

LOAD MOMENT ARM (mm)						

Molar	0.25	0.20	0.34	0.46	0.37	0.62
Incisal	0.44	0.35	0.60	0.49	0.39	0.66

MECHANICAL ADVANTAGE						
Molar position						

Superficial masseter	0.00	0.00	0.01	0.01	0.01	0.01
Anterior deep masseter	0.00	0.00	0.00	0.00	0.00	0.01
Posterior deep masseter	0.00	0.00	0.00	0.01	0.01	0.02
Anterior temporal	0.00	0.00	0.00	0.00	0.00	0.01
Posterior temporal	0.00	0.00	0.00	0.00	0.00	0.01
Medial pterygoid	0.01	0.01	0.01	0.01	0.01	0.02
Lateral pterygoid	0.00	0.00	0.01	0.01	0.01	0.02

Incisal position						

Superficial masseter	0.00	0.00	0.00	0.00	0.00	0.01
Anterior deep masseter	0.00	0.00	0.00	0.00	0.00	0.00
Posterior deep masseter	0.00	0.00	0.00	0.01	0.01	0.01
Anterior temporal	0.00	0.00	0.00	0.00	0.00	0.00
Posterior temporal	0.00	0.00	0.00	0.00	0.00	0.00
Medial pterygoid	0.00	0.00	0.01	0.01	0.01	0.01
Lateral pterygoid	0.00	0.00	0.00	0.01	0.01	0.01

*Left side*						
MUSCLE MOMENT ARM (mm)						

Superficial masseter	0.42	0.34	0.57	0.66	0.52	0.89
Anterior deep masseter	0.19	0.15	0.25	0.31	0.25	0.42
Posterior deep masseter	0.37	0.30	0.50	0.58	0.46	0.78
Anterior temporal	0.21	0.17	0.29	0.29	0.23	0.39
Posterior temporal	0.19	0.15	0.26	0.31	0.24	0.41
Medial pterygoid	0.24	0.19	0.32	0.66	0.53	0.89
Lateral pterygoid	0.30	0.24	0.40	0.96	0.76	1.29

LOAD MOMENT ARM (mm)						

Molar	0.31	0.25	0.42	0.42	0.33	0.56
Incisal	0.42	0.34	0.57	0.48	0.38	0.65

MECHANICAL ADVANTAGE						
Molar position						

Superficial masseter	0.01	0.01	0.01	0.01	0.01	0.01
Anterior deep masseter	0.00	0.00	0.00	0.00	0.00	0.01
Posterior deep masseter	0.00	0.00	0.01	0.01	0.01	0.01
Anterior temporal	0.00	0.00	0.00	0.00	0.00	0.01
Posterior temporal	0.00	0.00	0.00	0.00	0.00	0.01
Medial pterygoid	0.00	0.00	0.00	0.01	0.01	0.01
Lateral pterygoid	0.00	0.00	0.01	0.01	0.01	0.02
Incisal position						

Superficial masseter	0.00	0.00	0.01	0.01	0.00	0.01
Anterior deep masseter	0.00	0.00	0.00	0.00	0.00	0.00
Posterior deep masseter	0.00	0.00	0.00	0.01	0.00	0.01
Anterior temporal	0.00	0.00	0.00	0.00	0.00	0.00
Posterior temporal	0.00	0.00	0.00	0.00	0.00	0.00
Medial pterygoid	0.00	0.00	0.00	0.01	0.00	0.01
Lateral pterygoid	0.00	0.00	0.00	0.01	0.01	0.01

MME: Method of moments variance estimator.

## 4 Experimental Design, Materials and Methods

Thirty craniofacial cone-beam computed tomographies from healthy dentate voluntaries (12–19 age range, 16 female) were employed. A NewTom 3G Volumetric Scanner (Aperio Services, Verona, VR, Italy) with 12-in field of view, 8-mm aluminum filtration at 110 kV and 6.19 mAs, and slice thickness of 0.5 mm was used. Using the NewTom software 2.04 (Aperio Services), image data were converted to DICOM format with a voxel size of 0.25 mm. This study was supported by the Health Research Ethics Board of the University of Alberta (#5563), and all voluntaries signed written informed consent to participate.

### 4.1 Identification of landmarks

Through 390 to 430 DICOM slices, a three-dimensional body was constructed using the digital tools of Avizo Fire software 8.1 (Mercury Computer Systems, Inc., Berlin, BE, Germany). This program is a system for three-dimensional visualization, geometric reconstruction, and data analysis. For this purpose, the Isosurface function was used for image rendering. The view threshold was configured between −1000 and 2000. Within this range, a point around 600–650 was used for object visualization. This threshold provided the required specific density for the tomographic slides of bone structure to visualize in a three-dimensional image. As the threshold number increases, the density of bone structure observed decreases. By using the software's functions Orthoslice and Slide, the object was manipulated in various planes for convenience by adjusting the settings. These tools enabled the rotation and spatial alignment of the image. The colourmap properties were determined in setting number between −1000 and 4180. The contrast of the image was adjusted in the centre to ensure that all features are appropriately visible, while keeping the brightness constant. Therefore, using a precision mouse, it was possible to scroll up and down through the several patients' 2D slice images. By using the orientation properties, we can feasibly navigate through each chosen view of the skull. The orientation was defined with the *xz* plane as coronal, the *yz* plane as sagittal, and the *xy* plane as axial.

**Fig. 1:**
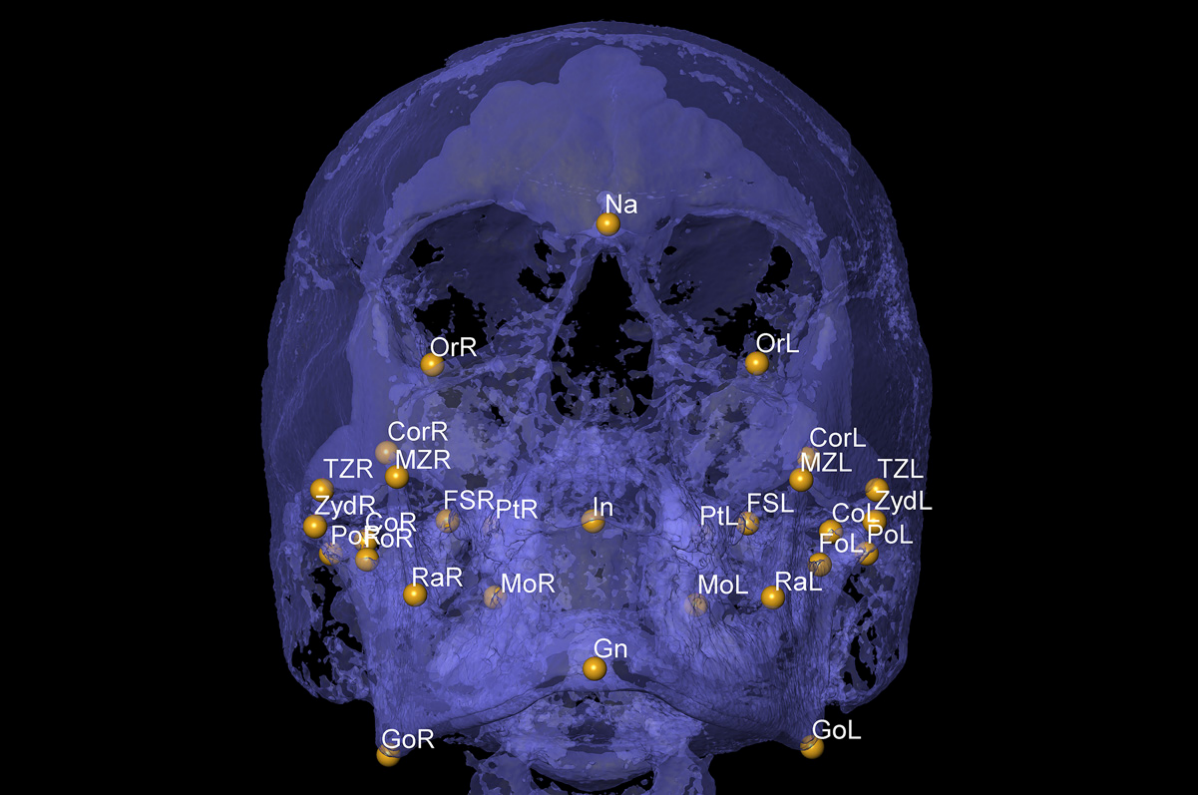
Coronal view for Nasion (Na), Gnathion (Gn) and Incisal (In) on the mid line, and Foramen spinosum (FS), Orbitale (Or), Porion (Po), Condylion (Co), Gonion (Go), Coronoid (Cor), Ramus (Ra), Maxillozygomatic suture (MZ), Temporozygomatic suture (TM), Pterygoid fovea (Fo), Pterygoid (Pt), Zyd and Molar (Mo) on the right (R) and left (L) sides.

**Fig. 2:**
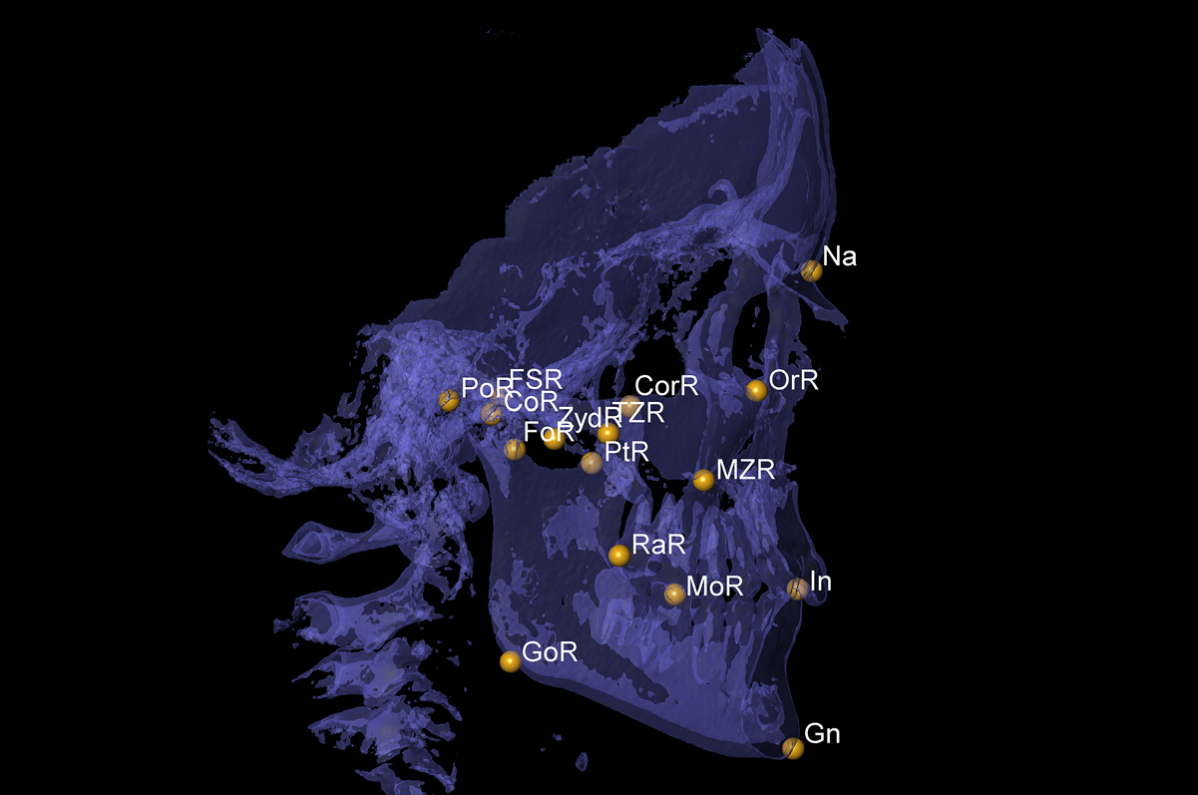
Sagittal view for Nasion (Na), Gnathion (Gn) and Incisal (In) on the mid line, and Foramen spinosum (FS), Orbitale (Or), Porion (Po), Condylion (Co), Gonion (Go), Coronoid (Cor), Ramus (Ra), Maxillozygomatic suture (MZ), Temporozygomatic suture (TM), Pterygoid fovea (Fo), Pterygoid (Pt), Zyd and Molar (Mo) on the right (R) and left (L) sides.

**Fig. 3:**
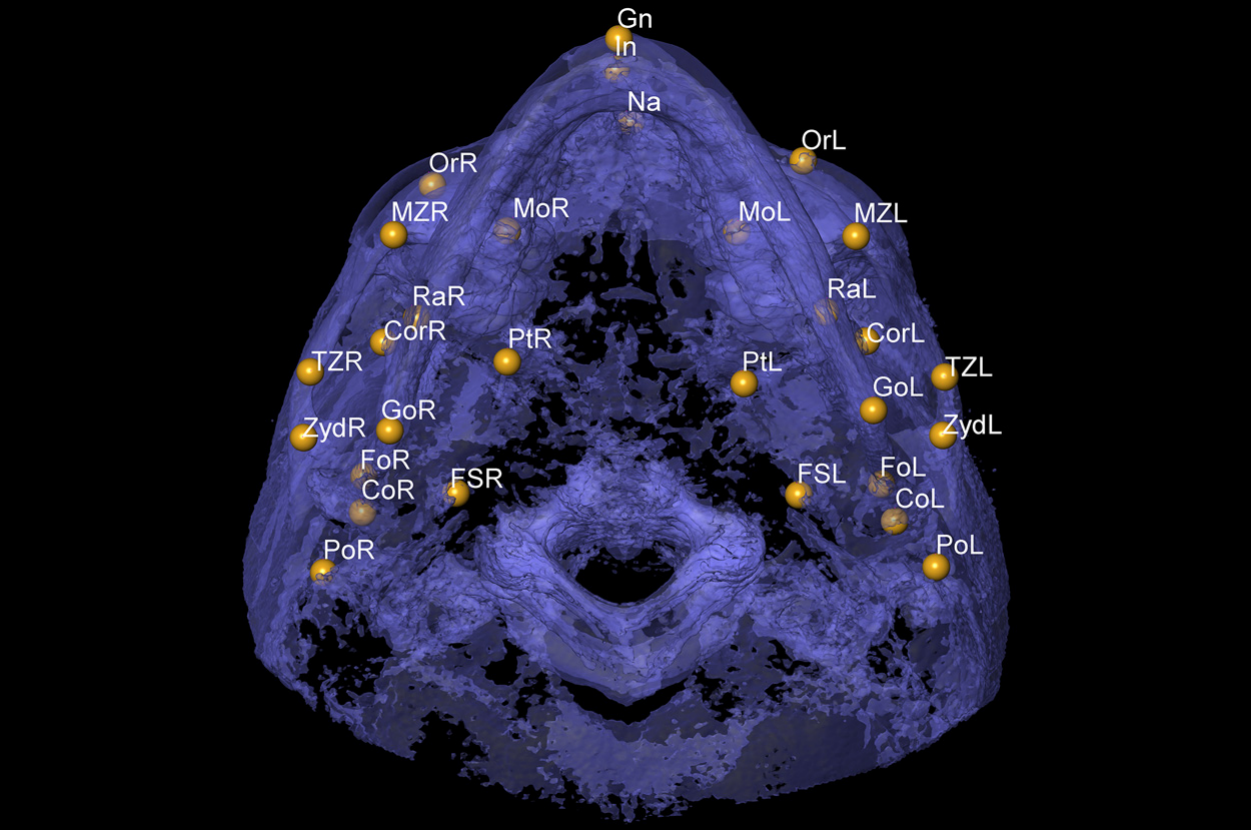
Axial view for Nasion (Na), Gnathion (Gn) and Incisal (In) on the mid line, and Foramen spinosum (FS), Orbitale (Or), Porion (Po), Condylion (Co), Gonion (Go), Coronoid (Cor), Ramus (Ra), Maxillozygomatic suture (MZ), Temporozygomatic suture (TM), Pterygoid fovea (Fo), Pterygoid (Pt), Zyd and Molar (Mo) on the right (R) and left (L) sides.

The landmark function added reference points for characterizing and simplifying various structures of interest. The size and spherical complexity of the landmarks was 1. Then, primary landmarks for Nasion (most anterior point of the frontonasal suture in the median plane), Gnathion (most anteroinferior point on the symphysis of the chin) and Incisal (junction of the borders of lower central incisors) positions were identified on the midline. Landmarks for Foramen spinosum (geometric center of the smallest circumference with the clearest defined borders viewed in axial view on the foramen spinosum), Orbitale (lowest point in the inferior margin of the orbit), Porion (superior point of the external auditory meatus), Condylion (most superior point on the condylar head), Gonion (point of intersection of the ramus plane and the mandibular plane), Coronoid (apex of the coronoid process), Ramus (point of intersection between the anterior border of the mandibular ramus and the mandible body), Maxillozygomatic (lower point of the maxillozygomatic suture), Temporozygomatic (lower point of the temporozygomatic suture), Pterygoid fovea (most concave point of the neck of the condyle), Pterygoid (midpoint of the lateral lamina of pterygoid process), Zyd (lowest point of articular tubercle), and Molar (central fossa of the lower first molar) locations were also bilaterally determined (Fig. 1, Fig. 2, Fig. 3) [7].

### 4.2 Transfer of landmark coordinates

The coordinates (*x, y,* and *z*) of the primary landmarks were transferred to the Cabri 3D software 2.1.2 (Cabrilog, Grenoble, Isère, France) to create secondary landmarks. This system is an interactive spatial geometry and mathematics program designed to explore the properties of dynamic bodies and create numerical constructions. The original values were reversibly divided proportionally by 10, due to the program's smaller visual field. By using the Point function, ELSA (midpoint on the line connecting both Foramen spinosum landmarks) was placed in the midline, and Maa (point that divides the anterior and middle thirds of the line connecting Ramus and Gonion), Map (point dividing the middle and posterior thirds of the line connecting Ramus and Gonion) and Zys (point that divides the posterior and middle thirds of the line joining Maxillozygomatic and Temporozygomatic) positions, on both sides [8]. With the use of Triangle function, the Frankfort (joining the right Porion and left Porion with the right Orbitale or left Orbitale), mandibular (joining the right Gonion and left Gonion with Gnathion) and sagittal (joining ELSA, Nasion, and Gnathion) planes were defined on the basis of the landmarks previously described. Moreover, accessory bilateral planes were oriented in the coronal (*xz*) and sagittal (*yz*) directions and constructed from the coronoid as references for the anterior and posterior temporal muscles. Landmarks and planes were used as regional references for muscle attachments to bones.

### 4.3 Mechanical advantage analysis

**Fig. 4:**
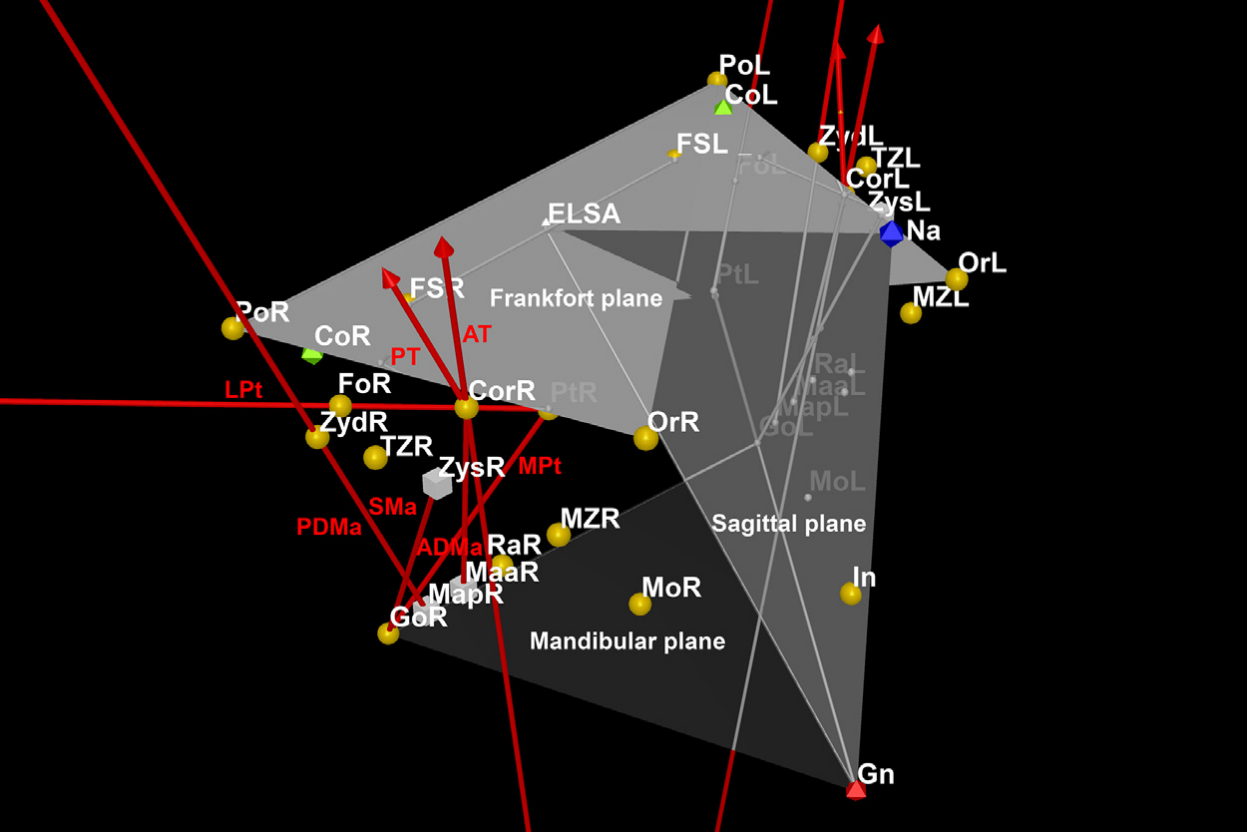
Muscle force vectors to the superficial masseter (SMa), anterior deep masseter (ADMa), posterior deep masseter (PDMa), anterior temporal (AT), posterior temporal (PT), medial pterygoid (MPt) and lateral pterygoid (LPt) muscles are presented as red lines in a right diagonal view.

Muscle force vectors were drawn using the Vector function to the superficial masseter (origin in Zys and insertion in Gonion), anterior deep masseter (origin in Coronoid and insertion in Maa), posterior deep masseter (origin in Zyd and insertion in Map), anterior temporal (origin in temporal fossa and insertion in Coronoid), posterior temporal (origin in temporal fossa and insertion in Coronoid), medial pterygoid (origin in Pterygoid and insertion in Gonion), and lateral pterygoid (origin in Pterygoid and insertion in Pterygoid fovea) muscles (Fig. 4).

Since the temporal fossa lacks a defined anatomical characteristic, the orientation of the temporal muscles was determined based on two vectors with angulation in the sagittal and axial planes of 10° and 75°, respectively, to the anterior fibres, and 35° and 61°, respectively, to the posterior fibres.

**Fig. 5:**
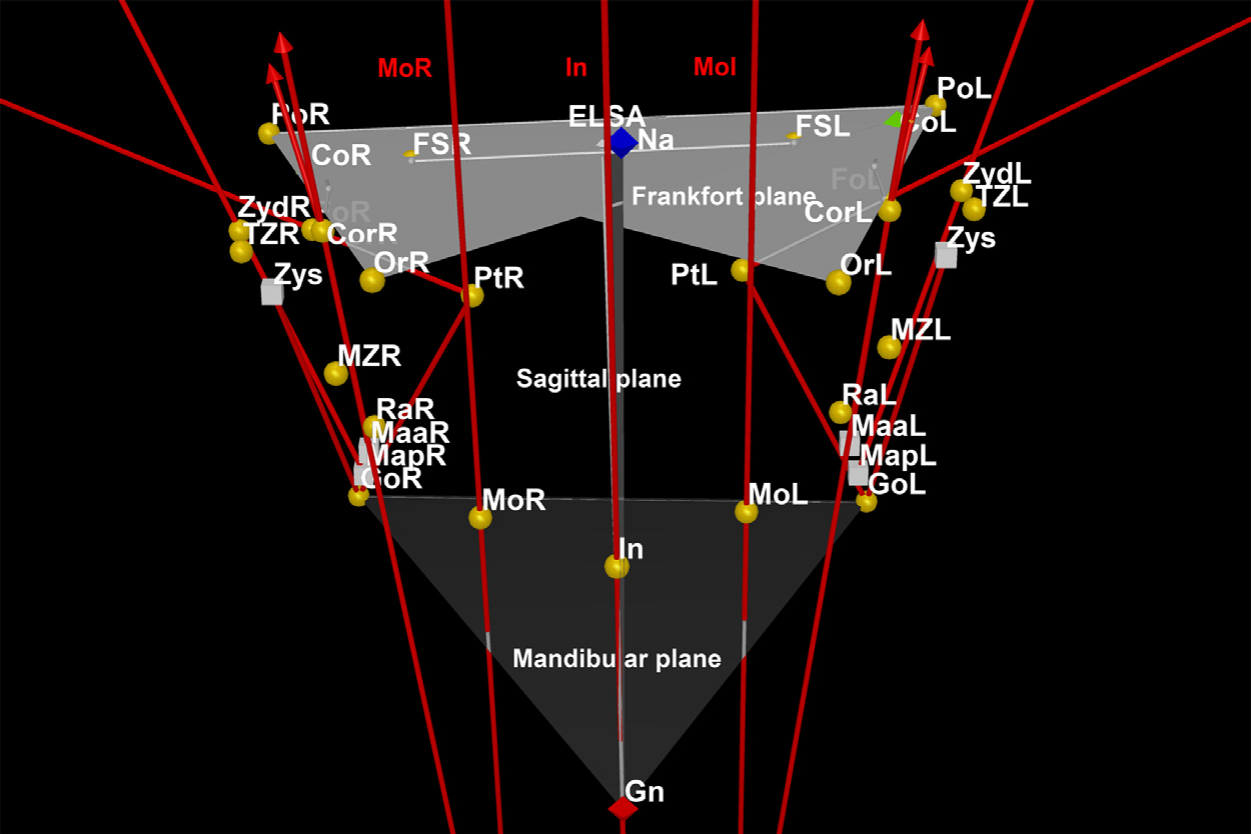
Bite force vectors at the molar (Mo) and incisal (In) positions on the right (R) and left (L) sides are displayed as red lines in a frontal superior view.

Bite force vectors were designed at right angles (Perpendicular function) to mandibular plane at the molar and incisal positions (Fig. 5).

**Fig. 6:**
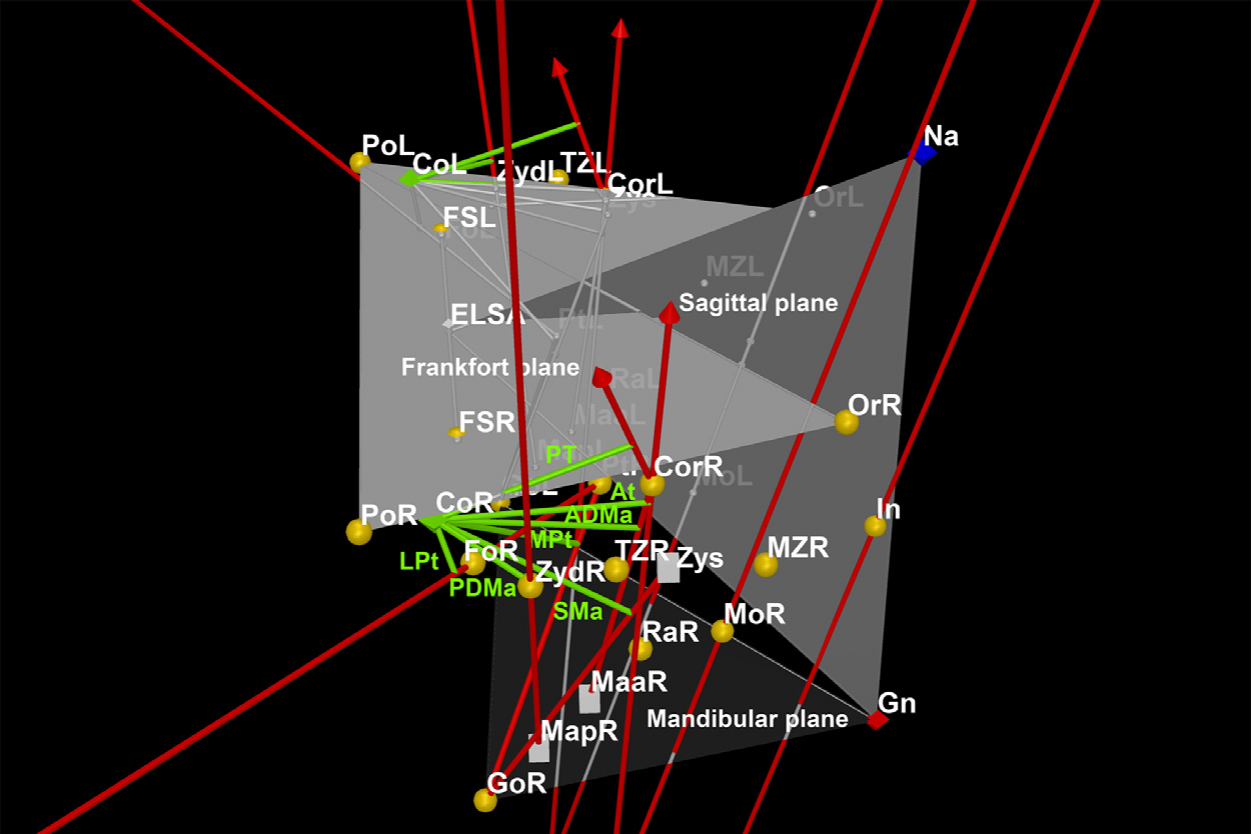
Muscle moment arms for the superficial masseter (SMa), anterior deep masseter (ADMa), posterior deep masseter (PDMa), anterior temporal (AT), posterior temporal (PT), medial pterygoid (MPt) and lateral pterygoid (LPt) muscles are presented as green lines in a right lateral view.

Moment arms to each muscle were then drawn as perpendicular lines (Segment function) to each muscle force vector from Condylion (Fig. 6). When that perpendicularity could not eventually be found, the muscle moment arms were adjusted to align with the projection of the muscle force vector (Line function).

**Fig. 7:**
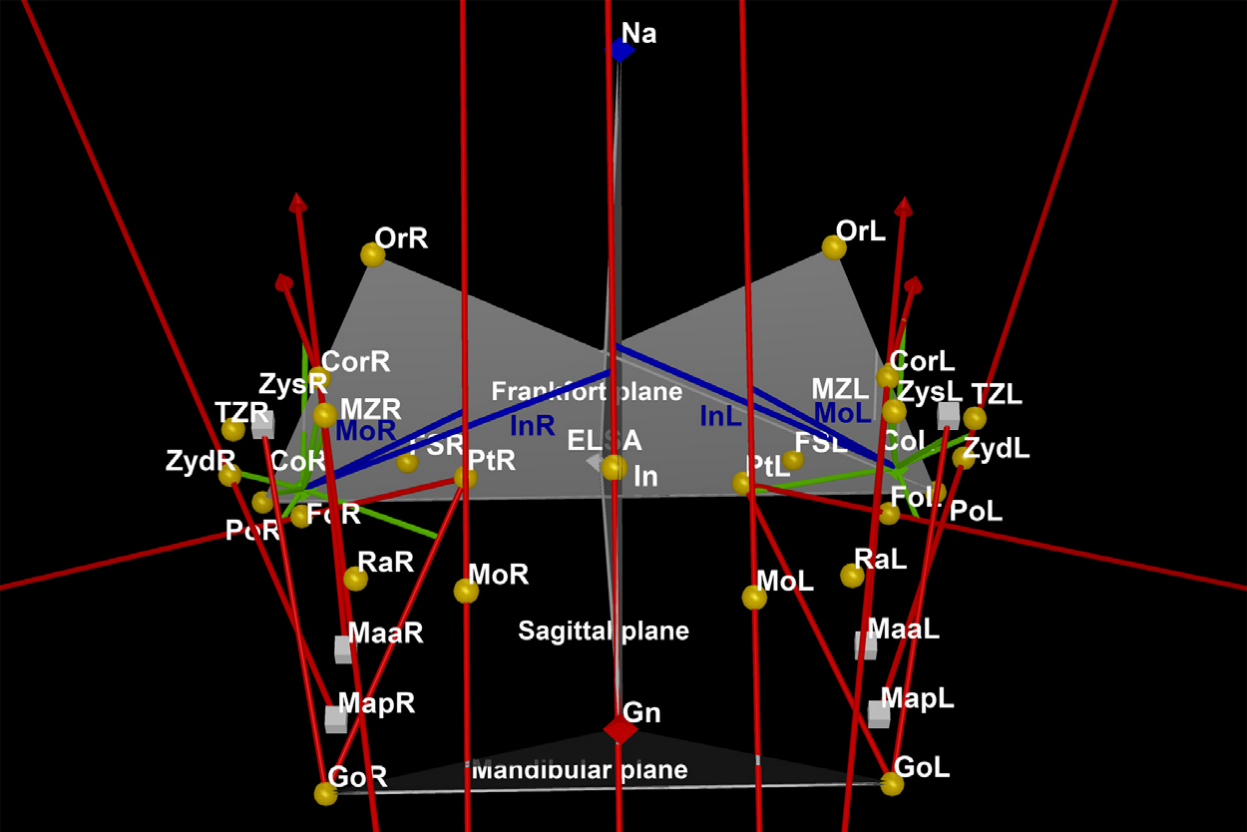
Load moment arms at the molar (Mo) and incisal (In) positions on the right (R) and left (L) sides are displayed as blue lines in a frontal inferior view.

Load moment arms were drawn as perpendicular segments to each bite force vector also from Condylion (Fig. 7).

All angulations were verified using the Angle function of the program.

**Fig. 8:**
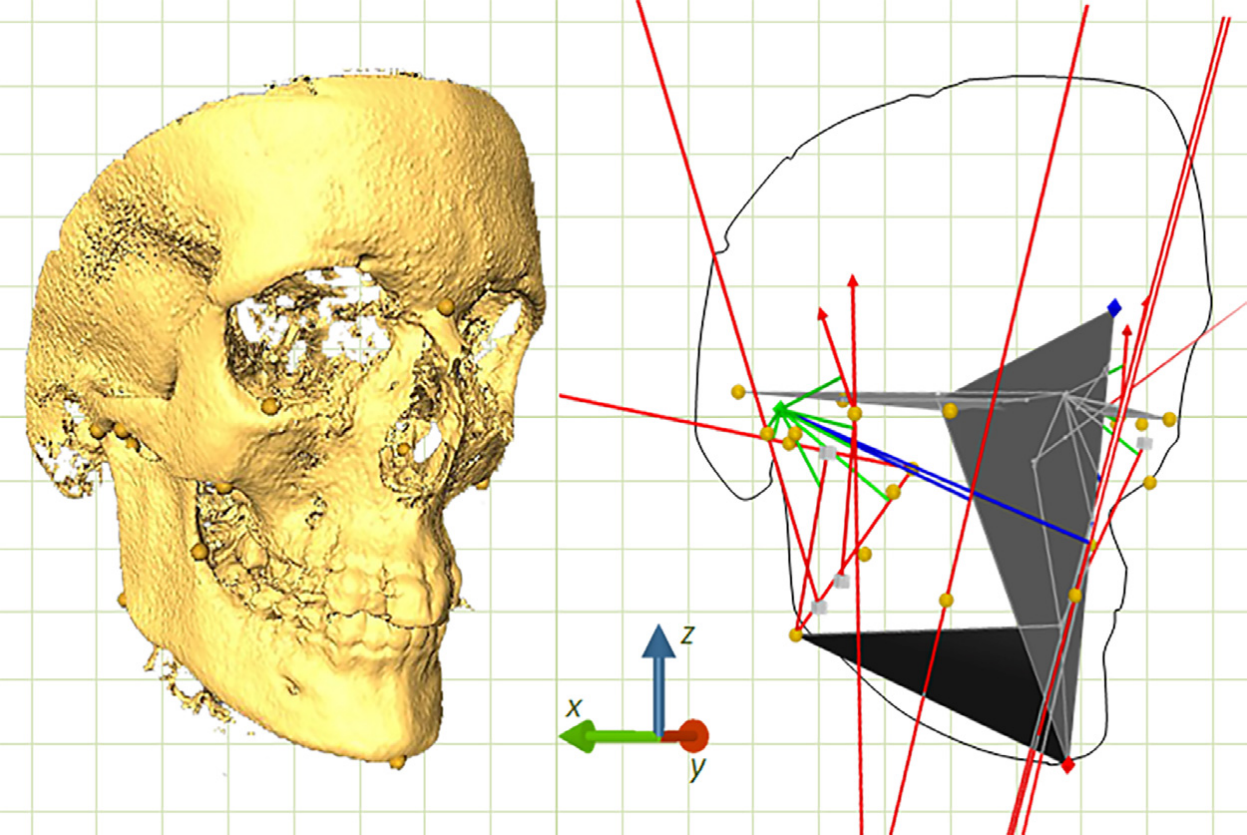
Complete model for all muscles.

Moment arms for all muscles were measured in mm using the Distance function, and the mechanical advantage was defined as the ratio of the muscle moment arm to the load moment arm (Fig. 8) [6]. Since it is a proportion, the transformation previously done for the coordinates (/10) had no effect on its own. However, the values were presented in their original magnitude (Table 3).

### 4.4 Precision analysis

A primary examiner repeated all measurements three times (trials 1, 2, and 3) after each 24-h interval. Two other examiners carried out these measures only once (trials 4 and 5, respectively). Cephalometric landmarks' coordinates (*x, y,* and *z*), muscle and load moment arm lengths (mm), and mechanical advantage ratios for the masticatory muscles were compared using the Friedman test (*α* = 0.05). Random errors were assessed by the square root of the ‘method of moments’ variance estimator.

## Limitations

In this precision research, the variations among subjects are more significant than the central tendency measures of the variables in the sample under study. Dentate subjects of various ages, races, and craniofacial characteristics were included. However, it would have been very interesting to determine the normative values of mandibular mechanical advantage, which could have been calculated with a larger and more homogeneous sample.

Given that the present analysis provides a plausible description of mandibular biomechanics, it is essential to have sufficient knowledge of craniofacial bone and muscular anatomy for image manipulation and landmark identification, as well as a basic understanding of spatial geometry.

## CRediT authorship contribution statement

**Dominique Ellen Carneiro:** Validation, Investigation, Writing – original draft. **Giancarlo De La Torre Canales:** Methodology, Resources, Writing – review & editing. **Manuel Óscar Lagravère:** Methodology, Resources, Writing – review & editing. **Nara Hellen Campanha:** Validation, Investigation. **Vanessa Migliorini Urban:** Validation, Investigation. **Alfonso Sánchez-Ayala:** Conceptualization, Methodology, Formal analysis, Writing – original draft, Writing – review & editing, Supervision, Project administration.

## Data Availability

Mechanical Advantage Raw Data (Original data) (Mendeley Data).

## Appendix Supplementary materials

Image, application 1
