# Supplementary material for: Trial data for precision analysis of a three-dimensional mandibular mechanical advantage
Source: Data Brief. 2024 Apr 10;54:110402. doi: 10.1016/j.dib.2024.110402 (PMC11043836; doi:10.1016/j.dib.2024.110402)
Supplement: Supplementary file 1 [file mmc1.pdf]

J Orofac Orthop (2023) 84:321–339
https://doi.org/10.1007/s00056-022-00378-7

ORIGINAL ARTICLE

A three-dimensional method to calculate mechanical advantage in mandibular function

Intra- and interexaminer reliability study

Alejandro Sánchez-Ayala[1] · Alfonso Sánchez-Ayala[1] · Rafaela Cristina Kolodzejezyk[1] · Vanessa Migliorini Urban[1] · Manuel Óscar Lagravère[1] · Nara Hellen Campanha[2]

Received: 20 June 2021 / Accepted: 16 January 2022 / Published online: 7 March 2022
© Springer Medizin Verlag GmbH, ein Teil von Springer Nature 2022

Abstract
Purpose Masticatory muscles are physically affected by several skeletal features. The muscle performance depends on muscle size, intrinsic strength, fiber direction, moment arm, and neuromuscular control. To date, for the masticatory apparatus, only a two-dimensional cephalometric method for assessing the mechanical advantage, which is a measure for the ratio of the output force to the input force in a system, is available. This study determined the reliability and errors of a three-dimensional (3D) mechanical advantage calculation for the masticatory system.
Methods Using cone-beam computed tomography images from teenage patients undergoing orthodontic treatments, 36 craniofacial landmarks were identified, and the moment arms for seven muscles and their load moment arms (biomechanical variables) were determined. The 3D mechanical advantage for each muscle was calculated. This procedure was repeated by three examiners. Reliability was verified using the intraclass correlation coefficient (ICC) and the errors by calculating the absolute differences, variance estimator and coefficient of variation (CV).
Results Landmark coordinates demonstrated excellent intra- and interexaminer reliability (ICC 0.998–1.000; $p<0.0001$). Intraexaminer data showed errors <1.5 mm. Unsatisfactory interexaminer errors ranged from 1.51–5.83 mm. All biomechanical variables presented excellent intraexaminer reliability (ICC 0.919–1.000, $p<0.0001$; CV<7%). Interexaminer results were almost excellent, but with lower values (ICC 0.750–1.000, $p<0.0001$; CV<10%). However, the muscle moment arm and 3D mechanical advantage of the lateral pterygoid muscles had ICCs<0.500 ($p<0.05$) and CV<30%. Intra- and interexaminer errors were ≤0.01 and ≤0.05, respectively.
Conclusions Both landmarks and biomechanical variables showed high reliability and acceptable errors. The proposed method is viable for the 3D mechanical advantage measure.

Keywords Masticatory muscles · Biomechanical phenomena · Cone-beam computed tomography · Teenagers · Mandible

✉ Prof. Dr. Alfonso Sánchez-Ayala, DDS MS PhD
snzcd@uepg.br

[1] Department of Dentistry, University of Ponta Grossa, Avenida General Carlos Cavalcanti n° 4748, Bloco M, Sala 64A, 84030-900 Ponta Grossa, Paraná, Brazil

[2] Orthodontic Graduate Program, University of Alberta, 116 St & 85 Ave, T6G 2R3 Edmonton, Alberta, Canada

Eine dreidimensionale Methode zur Berechnung des mechanischen Vorteils der Unterkieferfunktion

Studie zur Intra- und Inter-Untersucher-Reliabilität

Zusammenfassung

Zielsetzung Die Kaumuskulatur wird körperlich von mehreren Skelettmerkmalen beeinflusst. Die Muskelleistung hängt ab von der Muskelgröße, der intrinsischen Kraft, der Richtung der Muskelfasern, dem Impulsarm und der neuromuskulären Kontrolle. Bisher gibt es für den Kauorganapparat nur eine zweidimensionale kephalometrische Methode zur Beurteilung des mechanischen Vorteils, der ein Maß für das Verhältnis von Ausgangskraft zu Eingangskraft in einem System ist. In der vorliegenden Untersuchung wurden die Zuverlässigkeit und die Fehler einer dreidimensionalen (3-D) Berechnung des mechanischen Vorteils für den Kauapparat ermittelt.

Methoden Anhand von digitalen Volumentomographie-Aufnahmen jugendlicher Kieferorthopädie-Patienten wurden 36 kraniofaziale Orientierungspunkte identifiziert und die Momentenarme für 7 Muskeln und ihre Lastmomentarme (biomechanische Variablen) bestimmt. Der mechanische 3-D-Vorteil für jeden Muskel wurde berechnet. Dieses Verfahren wurde von 3 Untersuchern wiederholt. Die Zuverlässigkeit wurde anhand des Intraklassen-Korrelationskoeffizienten (ICC) überprüft, die Fehler durch Berechnung der absoluten Differenzen, des Varianzschätzers und des Variationskoeffizienten (CV).

Ergebnisse Die Koordinaten der Referenzpunkte wiesen eine ausgezeichnete Intra- und Inter-Untersucher-Reliabilität auf (ICC 0,998–1,000; $p<0{,}0001$). Die Intra-Untersucher-Daten zeigten Fehler <1,5 mm. Unbefriedigende Inter-Untersucher-Fehler reichten von 1,51-5,83 mm. Alle biomechanischen Variablen wiesen eine ausgezeichnete Intra-Untersucher-Reliabilität auf (ICC 0,919–1,000, $p<0{,}0001$; CV<7%). Die Inter-Untersucher-Ergebnisse waren fast hervorragend, jedoch mit niedrigeren Werten (ICC 0,750–1,000, $p<0{,}0001$; CV<10%). Der Drehmomentarm des Muskels und der mechanische 3-D-Vorteil der seitlichen Pterygoidmuskeln wiesen jedoch ICCs<0,500 ($p<0{,}05$) und CV < 30% auf. Intra- und Inter-Untersucher-Fehler waren ≤0,01 bzw. ≤0,05.

Schlussfolgerungen Sowohl die Referenzpunkte als auch die biomechanischen Variablen zeigten eine hohe Reliabilität und akzeptable Fehler. Die hier vorgeschlagene Methode ist für die Messung des mechanischen 3-D-Vorteils geeignet.

Schlüsselwörter Kaumuskeln · Biomechanische Phänomene · Digitale Volumentomographie · Jugendliche · Unterkiefer

Background

The locomotor apparatus can be simplified as a lever and rotation axes system. A lever is a basic mechanism that increases the force and speed of movements, and depends on a rigid bar that is rotated about a fulcrum. The bar is a long bone, and the fulcrum is a joint where the bones are supported and stabilized [1]. In a lever, a force called effort is applied at one point on the bar in order to move an object, known as resistance, located at some other point on the bar [2]. The forces transmission determines the intensity and direction of a moment of force or torque. Then, two moment arms are designed: the muscle moment arm and the load moment arm, which are defined as the perpendicular distances from the fulcrum to muscle force vector and load force vector, respectively [3].

The effectiveness of a lever can be assessed by computing its mechanical advantage (MA) as the ratio of the muscle moment arm to the load moment arm. Three lever classes can be designed to preserve the input power and trade off forces against movement to obtain a desired amplification in the output force. In a class I lever, the effort and the resistance are on opposite sides of the fulcrum. Although it may have any MA, in the human body it typically acts with a mechanical advantage = 1, working at balancing or modifying the motion direction without magnifying either the effort or resistance magnitudes [1]. The function of the posterior neck muscles to stabilize the head on the atlanto-occipital joint and the anterior thigh muscles to the knee during extension on the patella are examples (Fig. 1a).

In a class II lever, the resistance is located between the effort (both on the same side) and the fulcrum. The distance from the fulcrum to the resistance is less than to the effort and, thus, the mechanical advantage is > 1, amplifying the torque created by the effort [1]. There are relatively few examples, but the rising onto the toes is one of them (Fig. 1a). In a class III lever, the effort is between the resistance (also on the same side) and the fulcrum, acting with a mechanical advantage < 1. To overcome resistance, much greater effort requires working over a small distance. However, the resistance is moved over a much greater distance in the same amount of time (increased speed of motion). This arrangement is observed in nearly all extremities joints (Fig. 1a; [3]).

Although the neuromuscular control of mandibular movements is complex, clenching or isometric biting can also be understood as a class III lever [2]. In theory, the fulcrum is represented by the temporomandibular joints

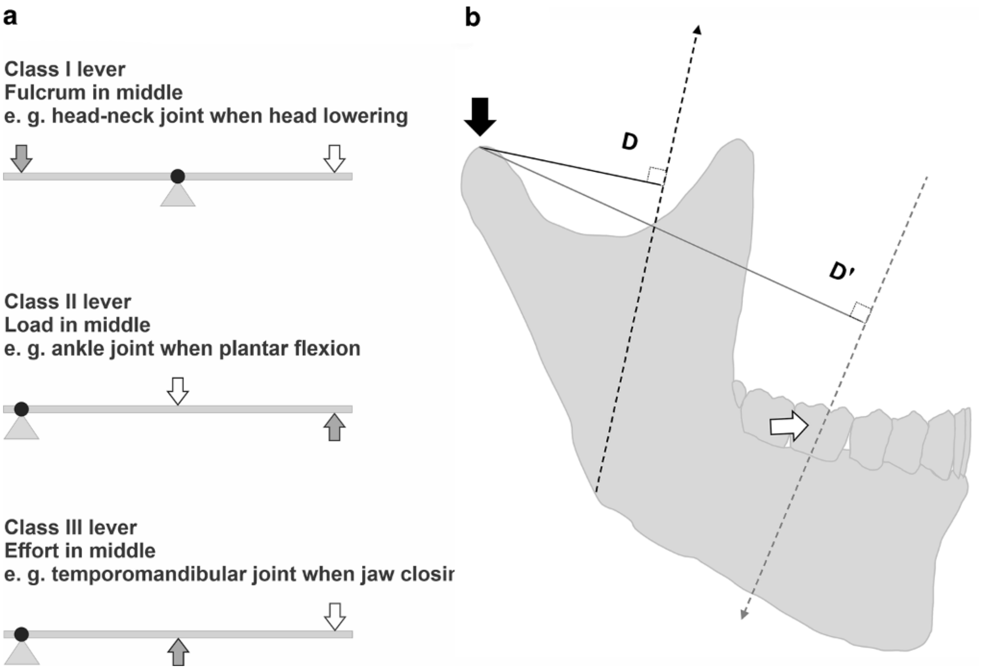

Fig. 1 **a** Three classes of lever systems, according to where effort (*gray arrow*) and resistance (*white arrow*) are located with respect to the fulcrum (*gray triangle*). **b** Basic two-dimensional mandibular lever system (for masseter muscle), where the muscle moment arm (*black line*) is designed as a perpendicular line to the muscle force vector (*black dashed line*) from the fulcrum, and the load moment arm (*gray line*) is drawn as a perpendicular line to the bite force vector (*gray dashed line*) also from the fulcrum. The mechanical advantage is then defined as the ratio of the moment arm distances (*D/D′*). The fulcrum (condyle) and bite position (molar tooth) are represented by *black and white arrows*, respectively
Abb. 1 **a** Drei Klassen von Hebelsystemen, entsprechend der Lage von Kraft (*grauer Pfeil*) und Widerstand (*weißer Pfeil*) in Bezug auf den Angelpunkt (*graues Dreieck*). **b** Grundlegendes zweidimensionales Unterkiefer-Hebelsystem (für den M. masseter), bei dem der Muskelmomentarm (*schwarze Linie*) als senkrechte Linie zum Muskelkraftvektor (*schwarze gestrichelte Linie*) vom Angelpunkt aus konstruiert ist und der Lastmomentarm (*graue Linie*) als senkrechte Linie zum Beißkraftvektor (*graue gestrichelte Linie*) gezeichnet ist, ebenfalls vom Angelpunkt aus. Der mechanische Vorteil ist dann definiert als das Verhältnis der Momentarmabstände (*D/D′*). Der Hebelpunkt (Kondylus) und die Bisslage (Molar) werden durch *schwarze* bzw. *weiße Pfeile* dargestellt

(TMJ), where the mandible freely rotates around them as a rigid bar. The effort is produced by the masticatory muscles, and the resistance is provided by the load on the teeth [4]. The muscle moment arm extends perpendicular from the TMJ to the muscle force vector, and the load moment arm, from the TMJ to the load or bite force vector (Fig. 1b; [5]). Therefore, its mechanical advantage being <1 favors the efficiency of the masticatory muscles in generating strength in a certain bite position for chewing food [6].

Despite the availability of more advanced methods in research, such as finite elements [7], a two-dimensional (2D) mechanical advantage approach using lateral radiographs has been considered for clinical studies of craniofacial growth changes [2, 4–6] and orthodontic treatment [8]. Unfortunately, this radiograph is limited due to multiple bone structures seen in a single view, bilateral overlapping by craniofacial asymmetry, distortion by different focal lengths, and use of a 2D analysis for three-dimensional (3D) structures [9]. Moreover, recognition of the muscle attachments for drawing the muscle force vectors is difficult and arbitrarily defined.

Changes of 3D mechanical advantage after orthognathic surgery has also been calculated using a series of magnetic resonance images (MRI) [10, 11]. However, it may be complicated to mathematically determine the muscle force vectors from the centroids of muscle segmentations using ordinary clinical procedures. Currently, the use of cone-beam computed tomography (CBCT) offers an integral visualization of craniofacial tissues [12]. A 3D method could identify anatomical landmarks more precisely and reliably, and establish spatial reference planes and plausible regions of origin and insertion of the masticatory muscles. With the help of a bilateral analysis, a more appropriate cephalometric assessment of the mechanical advantage becomes possible.

The 3D landmarks from CBCT are currently used for the diagnosis of anteroposterior [13] and transversal [14] malocclusions and to study skeletal and dental changes after orthodontic [15] and orthognathic surgery [16] and to analyze airways [17]. These reference points were conceptually derived or conveniently modified from classical cephalometric landmarks. Since previous analyses of mechanical advantage from lateral radiography also used these landmarks in

Table 1 Reference planes and landmarks
Tab. 1 Referenzebenen und Referenzpunkte

Variables	Definition	Function
Primary landmarks		
Nasion[a]	Most anterior point of the frontonasal suture in the median plane	Anterior reference for the sagittal plane
Gnathion[a]	Most anteroinferior point on the symphysis of the chin, constructed by intersecting a line drawn perpendicular to the line connecting menton and pogonion	Anterior reference for the sagittal and mandibular planes
Incisal[a]	Junction of the borders of lower central incisors	Reference for loading
Foramen spinosum	Geometric center of the smallest circumference with the clearest defined borders viewed in axial view on the foramen spinosum	Reference for ELSA identification
Orbitale	Lowest point in the inferior margin of the orbit	Anterior reference for the Frankfort plane
Porion	Superior point of the external auditory meatus	Posterior reference for the Frankfort plane
Condylion	Most superior point on the condylar head	Reference for arbitrary fulcrum
Gonion	Constructed point of intersection of the ramus plane and the mandibular plane	Reference for mandibular insertion of the superficial masseter and the medial pterygoid muscles, and for posterior reference of the mandibular plane
Coronoid	Apex of the coronoid process	Reference for mandibular insertion of the anterior deep masseter and the anterior temporal muscles
Ramus	Point of intersection between the anterior border of the mandibular ramus and the mandible body	Reference for mandibular insertion of the deep masseter muscles
Maxillozygomatic	Lower point of the maxillozygomatic suture	Anterior reference for zygomatic insertion of the superficial masseter muscle
Temporozygomatic	Lower point of the temporozygomatic suture	Posterior reference for zygomatic insertion of the superficial masseter muscle
Pterygoid fovea	Most concave point of the neck of the condyle	Reference for mandibular insertion of the lateral pterygoid muscle
Pterygoid	Midpoint of the lateral lamina of pterygoid process	Reference for pterygoid insertion of the medial and lateral pterygoid muscles
Zyd	Lowest point of articular tubercle	Reference for zygomatic insertion of the deep posterior masseter muscle
Molar	Central fossa of the lower first molar	Reference for loading
Secondary landmarks		
ELSA[a]	Midpoint on the line connecting both Foramen spinosum landmarks[b]	Posterior reference for the sagittal plane
Maa	Point that divides the anterior and middle thirds of the line connecting Ramus and Gonion	Reference for mandibular insertion of the anterior deep masseter muscle
Map	Point dividing the middle and posterior thirds of the line connecting Ramus and Gonion	Reference for mandibular insertion of the posterior deep masseter muscle
Zys	Point that divides the posterior and middle thirds of the line joining Maxillozygomatic and Temporozygomatic	Reference for zygomatic insertion of the superficial masseter muscle
Reference planes		
Frankfort plane[a]	Line joining the right Porion and left Porion with the right Orbitale or left Orbitale	Reference plane, perpendicular to accessory planes on coronal direction
Mandibular plane[a]	Line joining the right Gonion and left Gonion with Gnathion	Reference plane for the bite force vectors
Sagittal plane[a]	Line joining ELSA, Nasion, and Gnathion	Reference plane, parallel to accessory planes on sagittal direction
Accessories	Planes oriented on coronal (xz) and sagittal (yz) directions and constructed from Coronoid	References for the anterior and posterior temporal muscles

Landmark bilaterally located, except those designated with [a]
[b]ELSA is a personal name, and it was chosen because it is easy to locate by using the condyle and the glenoid fossa as guides and the most of the cranial base growth occurs in a child's first 5 years with only minor changes after that age [24]

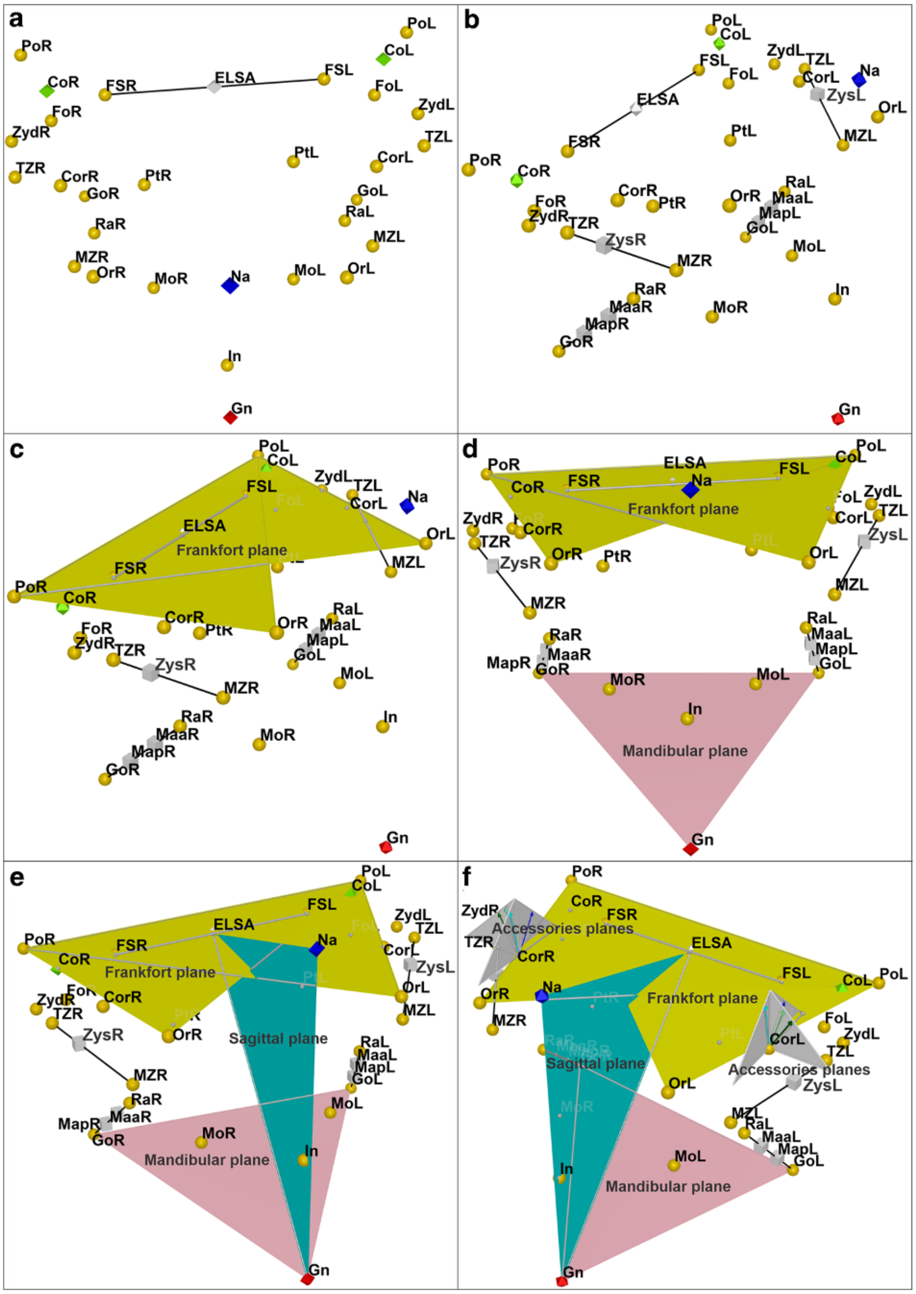

Fig. 2 Incisal on the midline, and Foramen spinosum, Orbitale, Porion, Gonion, Coronoid, Ramus, Maxillozygomatic suture, Temporozygomatic suture, Pterygoid fovea, Pterygoid, Zyd and Molar on the right (*R*) and left (*L*) sides are shown as spheres (*yellow*), in an anterior view. For easy identification and orientation, ELSA (between both Foramen spinosums united by a *black line*), Nasion and Gnathion on the midline, and Condylion on the R and L sides are exhibited as *diamonds* (*gray, blue, red* and *green*, respectively), in the same anterior view (**a**); Maa, Map (between Gonion and Ramus united by a *black line*) and Zys (between Maxillozygomatic suture and Temporozygomatic suture united by a *black line*) on the R and L sides are displayed as *squares (gray)*, in a right diagonal view (**b**). Frankfort plane (*yellow*) is text-indicated in a right diagonal view (**c**). Mandibular plane (*pink*) is text-indicated in an anterior superior view (**d**), Sagittal plane (*turquoise*) is text-indicated in a right diagonal view (**e**). Lateral accessory planes for the temporal muscle attachments (*gray*) are presented in a left diagonal view (**f**)

Abb. 2 „Inzisal“ auf der Mittellinie und Foramen spinosum, Orbitale, Porion, Gonion, Coronoid, Ramus, Sutura maxillozygomatica, Sutura temporozygomatica, Fovea pterygoidea, Pterygoidea, Zyd und Molar auf der rechten (*R*) und linken (*L*) Seite sind als Kugeln (*gelb*) in der anterioren Ansicht dargestellt. Zur einfachen Identifizierung und Orientierung sind ELSA (zwischen den beiden Foramina spinosa, die durch eine *schwarze Linie* verbunden sind), Nasion und Gnathion auf der Mittellinie und Kondylion auf der R und L Seite als *Rauten* (*grau, blau, rot* bzw. *grün*) in der gleichen Ansicht von vorne dargestellt (**a**); Maa, Map (zwischen Gonion und Ramus, die durch eine *schwarze Linie* verbunden sind) und Zys (zwischen Sutura maxillozygomatica und Sutura temporozygomatica, die durch eine *schwarze Linie* verbunden sind) auf der R und L Seite sind als *Quadrate* (*grau*) dargestellt, in einer Ansicht von rechts (**b**). Die Frankfurter Horizontale (*gelb*) ist in einer rechtsdiagonalen Ansicht (**c**) als Text dargestellt. Die Mandibularebene (*rosa*) ist in einer anterior-superioren Ansicht (**d**) dargestellt, die Sagittalebene (*türkis*) in einer rechts diagonalen Ansicht (**e**). Laterale akzessorische Ebenen für die temporalen Muskelansätze (*grau*) werden in einer links diagonalen Ansicht dargestellt (**f**)

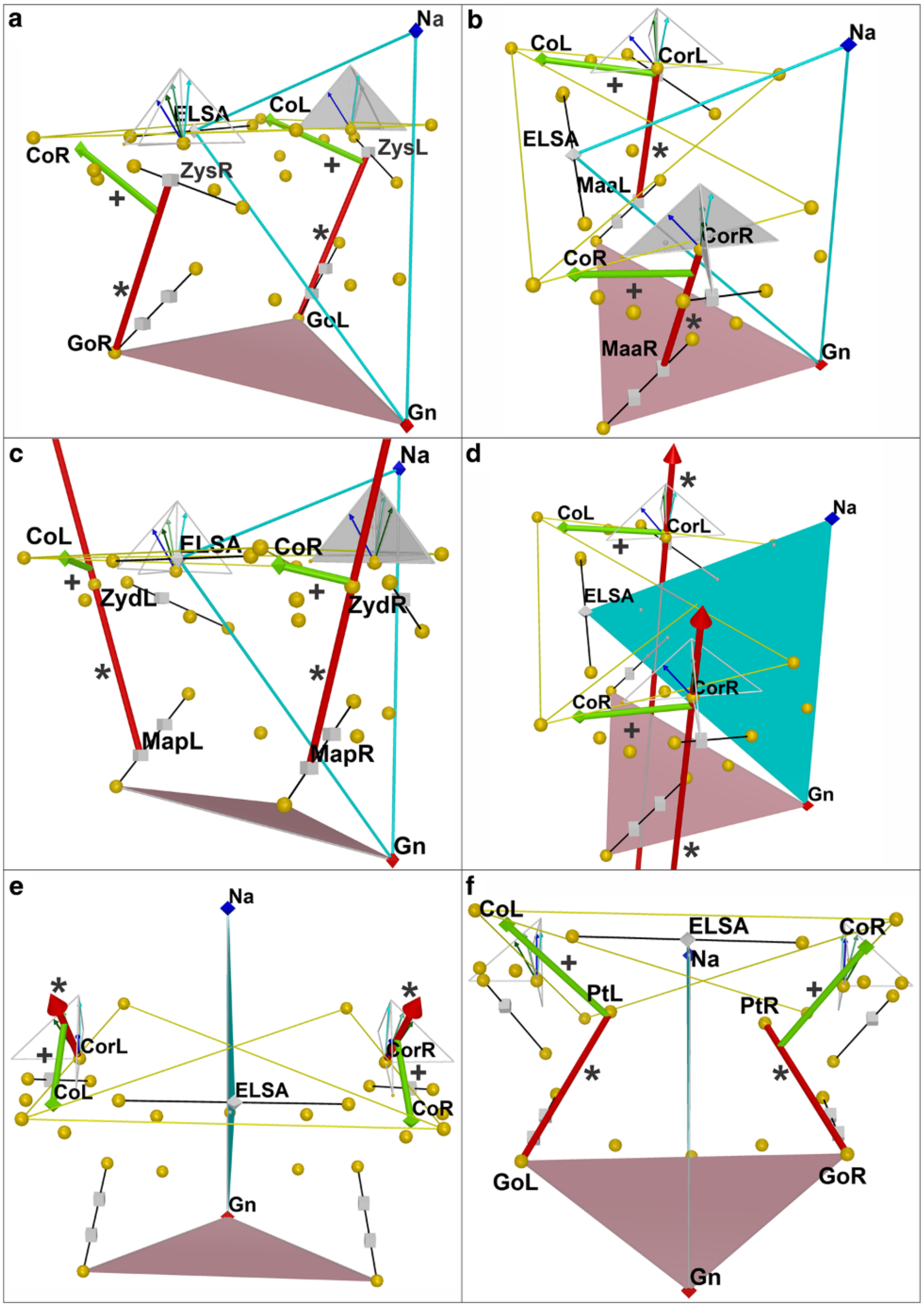

Fig. 3 ELSA, Nasion, Gnathion and Condylion are exhibited as *diamonds* (*gray, blue, red* and *green*, respectively). References planes Frankfort, Mandibular, Sagittal and Lateral accessories are displayed *transparent or not transparent* for better vision (*yellow, pink, turquoise* and *gray*, respectively). Muscle force vectors represented as *red lines marked with an asterisk*, and muscle moment arms denoted as *green lines marked with a plus sign* (from Condylion to muscle force vector), are shown for the superficial masseter, in a right diagonal view (**a**); anterior deep masseter, in a lateral superior view (**b**); posterior deep masseter, in a lateral posterior view (**c**); anterior temporal, in a lateral superior view (**d**); posterior temporal, in a posterior superior view (**e**); and medial pterygoid, in a posterior inferior view (**f**), on the right (*R*) and left (*L*) sides. The landmarks corresponding to each muscle attachment are shown in all views

Abb. 3 ELSA, Nasion, Gnathion und Condylion sind als *Rauten* dargestellt (*grau, blau, rot* bzw. *grün*). Die Referenzebenen Frankfurter Horizontale, Mandibular-, Sagittal- und Lateralebene sind zur besseren Sichtbarkeit *transparent* bzw. *nicht transparent* dargestellt (*gelb, rosa, türkis* bzw. *grau*). Muskelkraftvektoren, dargestellt als *rote und mit Sternchen markierte Linien*, Muskelmomentarme, dargestellt als *grüne und mit Pluszeichen markierte Linien* (vom Kondylion zum Muskelkraftvektor), sind für den M. masseter, pars superficialis, in einer rechten Diagonalansicht gezeigt (**a**); M. masseter anterior, in einer lateralen Ansicht von oben (**b**); M. masseter posterior, pars profunda, in einer lateralen Ansicht von hinten (**c**); M. temporalis anterior, in einer lateralen Ansicht von oben (**d**); M. temporalis posterior, in einer Ansicht von hinten oben (**e**); M. pterygoideus medialis, in einer Ansicht von hinten unten (**f**), auf der rechten (*R*) und linken (*L*) Seite. In allen Ansichten sind die zu jedem Muskelansatz gehörenden Referenzpunkte dargestellt

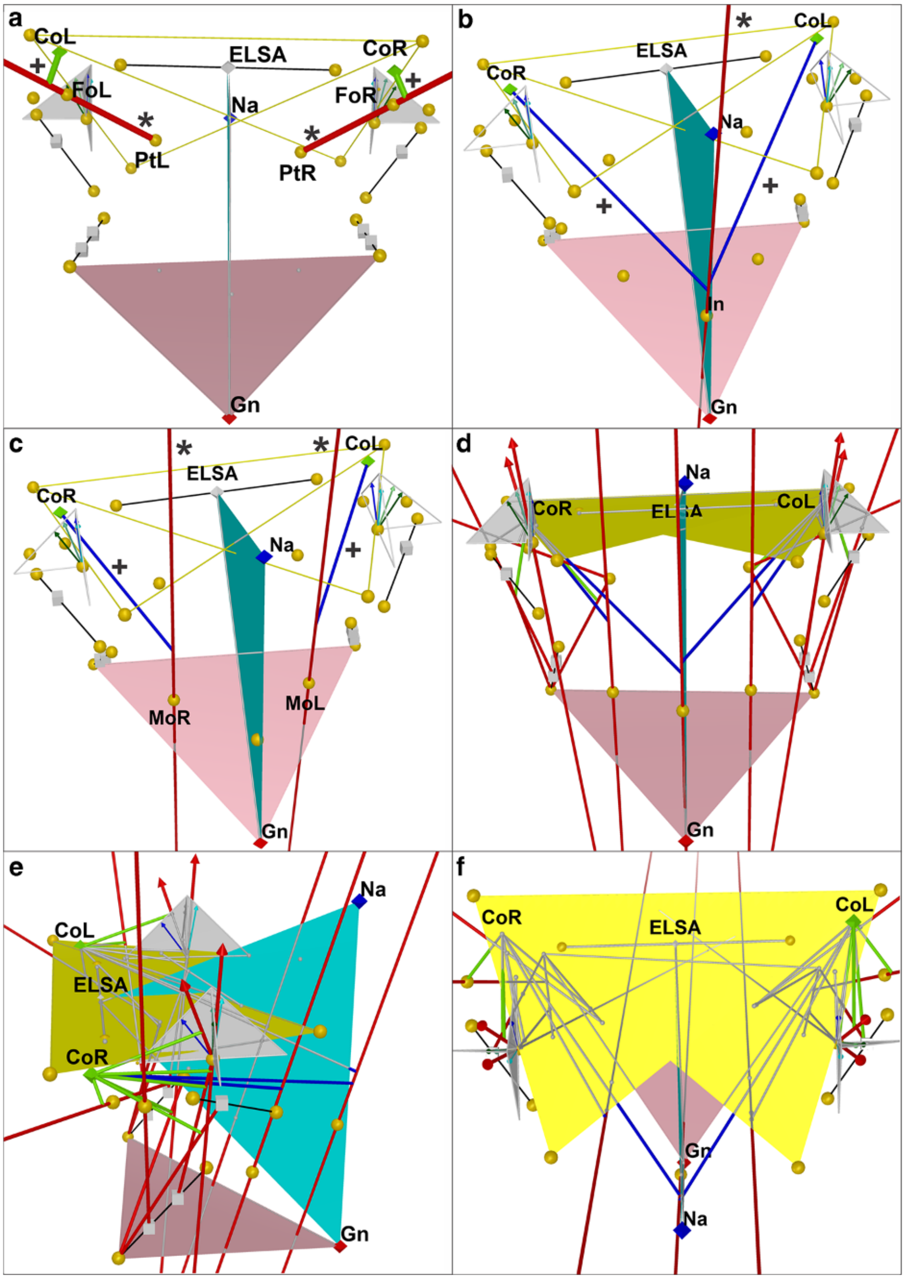

Fig. 4 ELSA, Nasion, Gnathion and Condylion are exhibited as *diamonds* (*gray, blue, red* and *green*, respectively). References planes Frankfort, Mandibular, Sagittal and Lateral accessories are displayed *transparent or not transparent* for a better vision (*yellow, pink, turquoise and gray*, respectively). Muscle force vectors represented as *red lines marked with an asterisk*, and muscle moment arms denoted as *green lines marked with a plus sign* (from Condylion to muscle force vector), are shown for the lateral pterygoid, in a posterior inferior view (**a**). Bite force vectors represented as *red lines marked with an asterisk*, and load moment arms denoted as *blue lines marked with a plus sign* (from Condylion to bite force vector), are shown at incisal (**b**) and molar (**c**) positions, in a right diagonal view. In the corresponding views the landmarks to each muscle attachment and bite positions are shown. Complete analysis is shown in anterior (**d**), lateral (**e**) and superior (**f**) views. It can be observed how all the muscular moment arms and load moment arms start from Condylion to each muscle and bite force vector, respectively

Abb. 4 ELSA, Nasion, Gnathion und Condylion sind als *Rauten* dargestellt (*grau, blau, rot* bzw. *grün*). Die Referenzebenen Frankfurter Horizontale, Mandibular-, Sagittal- und Lateralebene sind zur besseren Sichtbarkeit *transparent* bzw. *nicht transparent* dargestellt (*gelb, rosa, türkis* bzw. *grau*). Muskelkraftvektoren, dargestellt als *rote und mit Sternchen markierte Linien*, Muskelmomentarme, dargestellt als *grüne und mit Pluszeichen markierte Linien* (vom Kondylion zum Muskelkraftvektor), sind für den M. pterygoideus lateralis in einer posterioren Ansicht von unten gezeigt (**a**); Beißkraftvektoren, dargestellt als *rote und mit Sternchen markierte Linien*, und Lastmomentarme, dargestellt als *blaue und mit Pluszeichen markierte Linien* (vom Condylion zum Beißkraftvektor), sind in der Inzisal- (**b**) und Molarenposition (**c**) in einer rechten Diagonalansicht dargestellt. In den entsprechenden Ansichten sind die Referenzpunkte zu den einzelnen Muskelansätzen und Bisspositionen dargestellt. Die vollständige Analyse wird in der anterioren (**d**), lateralen (**e**) und superioren (**f**) Ansicht gezeigt. Zu beobachten ist, wie alle Drehmomentarme und Lastmomentarme der Muskeln vom Kondylion zu jedem Muskel- und Beißkraftvektor ausgehen

Table 2 Definition of muscular and bite force vectors
Tab. 2 Definition der Vektoren für muskuläre Kraft und Beißkraft

Biomechanical variables	Definition
Muscle force vectors	
Superficial masseter	Origin in Zys and insertion in Gonion
Anterior deep masseter	Origin in Coronoid and insertion in Maa
Posterior deep masseter	Origin in Zyd and insertion in Map
Anterior temporal	Origin in temporal fossa[a] and insertion in Coronoid
Posterior temporal	Origin in temporal fossa[b] and insertion in Coronoid
Medial pterygoid	Origin in Pterygoid and insertion in Gonion
Lateral pterygoid	Origin in Pterygoid and insertion in Pterygoid fovea
Bite force vectors	
Molar	Line perpendicular to the mandibular plane passing through Molar
Incisal	Line perpendicular to the mandibular plane passing through Incisal

[a]The arrangement of the anterior temporal muscle in the temporal fossa was determined as the result of two vectors with angulation of 10° and 75° in the sagittal and axial planes, respectively [26]
[b]The arrangement of the posterior temporal muscle in the temporal fossa was determined as the result of two vectors with angulation of 35° and 61° in the sagittal and axial planes, respectively [26]

2D space, we intended to improve the design of muscle and load moment arms by the using 3D landmarks.

For educational and research purposes, as well as for diagnosis and planning, it is fundamental to know how changes in position and size of the mandible and maxilla could alter the mechanical advantage. The muscle moment arms may change by the dislocation of muscular attachments, and the load moment arms through spatial movement of the occlusal plane and teeth positions [18]. Thereby, orthopedic or orthodontic functional appliances and orthognathic surgical guides could be designed for redirecting facial growth and the maxillomandibular relationship, respectively, to a favorable mechanical advantage after treatment. The aim of this study was to determine the intra- and interexaminer reliability of a 3D mechanical advantage method with the use of CBCT for seven mandibular muscles. We hypothesize that the evaluated elements from the proposed method (landmark coordinates recognition, muscle and bite force vector designs, muscle and load moment arm measurements, and mechanical advantage calculus) have satisfactory reliability and acceptable errors to be clinically applied.

Patients and methods

Sample

Thirty CBCTs from healthy dentate individuals (age 12–19 years; 16 females, 14 males) from the University of Alberta Orthodontic Program were used. These images were taken for diagnostic purposes for routine orthodontic records and not for this research. Subjects with systemic diseases, syndromes and previous orthodontic treatment were excluded. This work was approved by the Health Research Ethics Board at the University of Alberta (#5563), and all subjects signed written informed consent to participate. A minimum sample size ($n=17$) was calculated assuming three repetitions by different examiners and a minimum acceptable intraclass correlation coefficient (ICC) of 0.7 to expect an ICC of 0.9 ($\alpha=0.05$, $\beta=0.20$). However, as previously recommended [19] for error analysis, 30 CBCTs were used.

Cone-beam computed tomography acquisition

A NewTom 3G Volumetric Scanner (Aperio Services, Verona, Italy) was used with a 12-inch field of view with 8-mm aluminum filtration at 110 kV and 6.19 mA and slice thickness of 0.5 mm. Subjects were placed in the dorsal decubitus position with the Frankfort plane perpendicular to the floor. Raw image data were obtained from 390–430 slices and converted to DICOM format using the NewTom software 2.04 (Aperio Services) with a voxel size of 0.25 mm. Using Avizo Fire software 8.1 (Mercury Computer Systems, Inc., Berlin, Germany), the DICOM images were rendered into a volumetric image using 512 × 512 matrices. For orientation purposes, the planes were defined as the xz being coronal (frontal), yz sagittal (median, longitudinal), and xy axial (transversal, horizontal) [9]. The coordinate system and the origin (0, 0, 0) established by the software were used to identify craniofacial landmarks. Although the landmarks used were originally created in a 2D space for conventional cephalometry and rely on the overprojection from both sides of the skull, the use of these landmarks three-dimensionally has already been previously studied ([9, 20–23]; Table 1, Supplementary material Figs. 1–3; Figs. 2, 3 and 4; Supplementary material Video 1).

Mechanical advantage method

Primary landmarks for Nasion, Gnathion and Incisal positions were detected in the midline (Table 1, Supplementary material Fig. 1). Landmarks for Foramen spinosum, Orbitale, Porion (Table 1, Supplementary material Fig. 1), Condylion, Gonion, Coronoid, Ramus, Maxillozygomatic suture, Temporozygomatic suture (Table 1, Supplementary

Table 3 Intraexaminer errors of landmark coordinates (mm)
Tab. 3 Intra-Untersucher-Fehler bei den Koordinaten der Referenzpunkte (mm)

Landmarks	x		y		z	
	AMAD	SDMAD	AMAD	SDMAD	AMAD	SDMAD
ELSA	0.244	0.156	0.331	0.226	0.569	0.530
Nasion	0.228	0.140	0.154	0.146	0.440	0.496
Gnathion	0.372	0.222	0.504	0.338	0.422	0.326
Incisal	0.232	0.224	0.392	0.426	0.333	0.641
Right side						
Foramen spinosum	0.412	0.489	0.458	0.386	0.761	0.973
Orbitale	0.721	0.444	0.564	0.337	0.138	0.092
Porion	1.457	1.597	0.655	0.454	0.322	0.342
Condylion	0.339	0.248	0.218	0.104	0.102	0.325
Gonion	0.478	0.366	1.034	0.612	1.373	0.737
Coronoid	0.208	0.242	0.247	0.191	0.205	0.217
Ramus	0.427	0.287	0.450	0.355	1.086	0.636
Maxillozygomatic	0.943	1.338	0.581	0.546	0.496	0.466
Temporozygomatic	0.316	0.193	0.315	0.222	0.256	0.219
Pterygoid fovea	0.325	0.232	0.609	0.442	0.763	0.483
Pterygoid	0.612	0.425	0.880	0.775	0.923	0.670
Zyd	0.487	0.309	0.562	0.507	0.187	0.129
Molar	0.302	0.197	0.285	0.166	0.192	0.263
Maa	0.342	0.169	0.464	0.331	0.847	0.571
Map	0.351	0.237	0.720	0.441	1.011	0.579
Zys	0.424	0.419	0.287	0.183	0.278	0.213
Left side						
Foramen spinosum	0.308	0.248	0.305	0.231	0.553	0.442
Orbitale	0.837	0.567	0.639	0.631	0.166	0.123
Porion	1.273	1.136	0.638	0.503	0.447	0.420
Condylion	0.327	0.186	0.252	0.115	0.078	0.108
Gonion	0.521	0.408	0.952	0.726	1.383	0.880
Coronoid	0.190	0.170	0.244	0.199	0.180	0.125
Ramus	0.399	0.234	0.472	0.343	1.317	0.774
Maxillozygomatic	0.862	1.394	0.536	0.379	0.466	0.344
Temporozygomatic	0.461	0.500	0.603	1.356	0.434	0.310
Pterygoid fovea	0.447	0.324	0.478	0.310	0.732	0.439
Pterygoid	0.490	0.380	0.564	0.330	0.784	0.571
Zyd	0.534	0.390	0.452	0.330	0.226	0.157
Molar	0.343	0.406	0.396	0.372	0.334	0.489
Maa	0.318	0.156	0.513	0.338	1.111	0.639
Map	0.367	0.254	0.691	0.518	1.138	0.694
Zys	0.538	0.566	0.511	0.911	0.369	0.265

AMAD Average mean of absolute differences, *SDMAD* Standard deviation mean of absolute differences

material Fig. 2), Pterygoid fovea, Pterygoid, Zyd and Molar (Table 1, Supplementary material Fig. 3) locations were bilaterally recognized (Supplementary material Video 1). The 3D coordinates from the landmarks were transferred to the Cabri 3D software 2.1.2 (Cabrilog, Grenoble, Isère, France) to create secondary landmarks for the ELSA position in the midline [24], and Maa, Map and Zys locations on both sides (Table 1, Fig. 2a, b). Moreover, the Frankfort, mandibular and sagittal planes were defined on the basis of the points previously described (Table 1, Fig. 2c–e).

Muscle force vectors were drawn according to regional bone attachments for the superficial masseter, anterior deep masseter, posterior deep masseter, anterior temporal, posterior temporal, medial pterygoid (Table 2, Fig. 3a–f), and lateral pterygoid muscles (Table 2, Fig. 4a; [1, 2, 4–6, 8, 25–28]).

Bite force vectors were drawn perpendicular to the mandibular plane at the molar and incisal positions (Table 2, Fig. 4b, c).

Moment arms for each muscle were drawn as perpendicular lines to each muscle force vector (or their colinear projections, looking for perpendicularity) from Condylion (fulcrum; Figs. 3 and 4). Load moment arms were drawn as perpendicular lines to each bite force vector, also from Condylion (Fig. 4b–f). Moment arms for all muscles were measured (mm), and the mechanical advantage was calculated as the ratio of the muscle moment arm to the load moment arm for each set of landmarks collected (Supplementary material Video 1).

Study design and statistical analysis

This cross-sectional agreement study used five trials on the entire sample. The main examiner repeated all measurements three times (trials # 1, # 2, and # 3) each after a 24 h interval. Two other examiners carried out these measurements only once (trials # 4 and # 5, respectively). Intraexaminer reliability was evaluated by comparing results of the main examiner. Interexaminer reliability was verified by comparing trial # 1 (randomly selected) with trials # 4 and # 5. The main examiner had training on tomographic craniofacial anatomy, and he calibrated the other examiners. Examiners were blinded to identification and order of all assessments.

Reliability of measurements was determined using SPSS software (v.25, IBM, Armonk, NY, USA) considering an ICC two-factor mixed model with absolute agreement [29, 30]. ICC was considered excellent when ≥ 0.75, satisfactory between 0.40 and 0.74, and poor when < 0.40.

Errors were determined by the average mean of absolute differences $\left[\sum_{i=1}^{n}\left(|t_{1i}-t_{2i}|+|t_{1i}-t_{3i}|+|t_{2i}-t_{3i}|\right)\right]/3n$, where t is each trial and n is the sample size [20]. Random error was also estimated by the square root of the ‘method of moments’ variance estimator $S_M = \sqrt{\left(\sum_{i=1}^{n}(d_i-\overline{d})^2/2(n-1)\right)}$, where d is the mean of absolute differences, $\overline{d}$ is the average mean of the absolute difference, and n is the sample size [19].

To analyze how disagreements affected biomechanical metrics, the coefficient of variation was considered as the percentage ratio between the standard deviation and mean of measurements. Therefore, the relative dispersion of data was calculated from the three inter- and intraexaminer trials [31].

Results

Reliability of landmark coordinates

Excellent intra- and interexaminer reliability were found for all landmark coordinates, with ICC values ranging from 0.998 to 0.999 ($p < 0.0001$).

Errors in landmark coordinates

Intraexaminer errors for the average mean of absolute differences and the method of moments variance estimator were less than 1.5 mm (Table 3).

However, interexaminer errors for the average mean of absolute differences were higher (Table 4). On the right side, the landmark for Orbitale presented with errors of 4.681 mm, 3.254 mm, and 1.791 mm in the x, y, and z coordinates, respectively. Porion showed an error of 2.152 mm in the x-axis. Errors of 1.732 mm and 1.649 mm for Gonion, 2.147 mm and 3.798 mm for Ramus, 1.631 mm and 1.802 mm for Pterygoid, 1.920 mm and 2.878 mm for Maa, and 1.749 mm and 2.056 mm for Map were found in the y and z coordinates, respectively. In the z axis corresponding to Pterygoid fovea, a 2.570 mm error was found (Table 4).

On the left side, Orbitale showed errors of 5.834 mm, 3.750 mm, and 2.083 mm in the x, y, and z coordinates, respectively. Ramus maintained errors of 1.643 mm, 2.465 mm, and 4.431 mm also in the three axes, respectively. Porion presented an error of 2.266 mm in the x-axis.

In the y and z coordinates, errors of 2.028 mm and 2.364 mm for Gonion, 1.512 mm and 2.093 mm for Pterygoid, 1.854 mm and 2.740 mm for Pterygoid fovea, 2.064 mm and 3.256 mm for Maa, and 1.891 mm and 2.478 mm for Map were seen.

For the method of moments variance estimator, results only indicated an interexaminer error of 1.55 mm (1.23–2.08, 95% confidence interval) for the right Porion x-axis.

Coefficients of variation for the mechanical advantage measurements

Intra- and interexaminer data for the mechanical advantage for the five sets of landmarks is shown in Table 5. A high agreement was found for the intraexaminer comparisons, determining a coefficient of variation < 3% for almost all biomechanical variables, with the exception of the variables related to the deep posterior masseter and lateral pterygoid muscles, which both increased by 3–7%, respectively (Table 5). This same trend was observed for the interexaminer calculations, where the variables related to these muscles showed values from 8–10% and from 21–29%, respectively.

Table 4 Interexaminer errors of landmark coordinates (mm)
Tab. 4 Inter-Untersucher-Fehler bei den Koordinaten der Referenzpunkte (mm)

Landmarks	x		y		z	
	AMAD	SDMAD	AMAD	SDMAD	AMAD	SDMAD
ELSA	0.422	0.269	0.687	1.107	0.807	0.556
Nasion	0.335	0.172	0.236	0.230	0.647	0.725
Gnathion	0.804	0.402	0.939	0.622	1.069	0.553
Incisal	0.478	0.722	0.700	0.432	0.511	0.645
Right side						
Foramen spinosum	0.642	0.433	0.613	0.490	1.051	0.770
Orbitale	**4.681**	1.608	**3.254**	1.258	**1.791**	0.934
Porion	**2.152**	2.187	1.118	0.836	0.527	0.441
Condylion	1.217	1.111	0.607	0.421	0.570	0.612
Gonion	0.801	0.457	**1.732**	1.337	**1.649**	1.000
Coronoid	0.563	0.486	0.621	0.452	0.369	0.253
Ramus	0.994	0.597	**2.147**	1.040	**3.798**	1.390
Maxillozygomatic	0.857	0.768	0.880	0.575	0.576	0.439
Temporozygomatic	0.681	0.374	0.584	0.446	0.478	0.290
Pterygoid fovea	1.006	0.610	1.348	0.650	**2.570**	1.353
Pterygoid	1.041	0.525	**1.631**	1.448	**1.802**	0.915
Zyd	0.558	0.540	0.869	0.693	0.301	0.226
Molar	1.032	0.786	0.828	0.764	0.514	0.409
Maa	0.542	0.389	**1.920**	0.865	**2.878**	1.052
Map	0.469	0.281	**1.749**	1.038	**2.056**	0.989
Zys	0.611	0.370	0.529	0.414	0.451	0.277
Left side						
Foramen spinosum	0.714	0.693	1.007	1.883	0.808	0.683
Orbitale	**5.834**	1.737	**3.750**	0.944	**2.083**	0.840
Porion	**2.266**	1.773	1.261	0.961	0.776	0.667
Condylion	1.474	1.020	0.832	0.453	0.516	0.468
Gonion	0.691	0.413	**2.028**	0.969	**2.364**	1.234
Coronoid	0.474	0.300	0.475	0.448	0.328	0.259
Ramus	**1.643**	0.842	**2.465**	1.157	**4.431**	1.536
Maxillozygomatic	1.339	2.036	0.880	0.572	0.665	0.851
Temporozygomatic	0.789	0.807	0.894	1.986	0.599	0.416
Pterygoid fovea	0.996	0.677	**1.854**	0.810	**2.740**	1.343
Pterygoid	1.120	0.609	**1.512**	1.095	**2.093**	1.114
Zyd	0.653	0.528	0.694	0.383	0.296	0.238
Molar	1.103	0.978	0.800	0.548	0.600	0.506
Maa	1.004	0.582	**2.064**	0.914	**3.256**	1.260
Map	0.529	0.322	**1.891**	0.838	**2.478**	1.178
Zys	0.733	0.645	0.709	1.296	0.460	0.341

Average values > 1.5 mm are shown in bold
AMAD Average mean of absolute differences, *SDMAD* Standard deviation mean of absolute differences

However, the other variables showed a coefficient of variation < 4% (Table 5).

Reliability of the mechanical advantage measurements

All biomechanical variables presented excellent reliability, showing ICC values ranging from 0.919–0.999 for the intraexaminer evaluation ($p < 0.0001$) (Table 6).

Almost all of the results of the interexaminer comparisons can also be considered excellent, even if presenting

with absolute lower values (0.750–1.000; $p < 0.0001$; Table 6). However, on the right side, the muscle moment arm and the 3D mechanical advantage for the lateral pterygoid muscle (molar position) revealed satisfactory (0.434) and poor (0.394) ICC values ($p < 0.01$), respectively. The 3D mechanical advantage at the incisal position of the medial and lateral pterygoid muscles also had satisfactory (0.720) and poor (0.397) ICCs ($p < 0.01$), respectively (Table 6). On the left side, the muscle moment arm, and the 3D mechanical advantage at molar and incisal positions of the lateral pterygoid muscle presented poor ICC values, with values of 0.313, 0.341, and 0.322 ($p < 0.01$), respectively (Table 6).

Errors in the mechanical advantage measurements

All intraexaminer errors for the average mean of absolute differences (Table 6) and the method of moments variance estimator were less than 1.0 mm.

On the right side, for the interexaminer average mean of absolute difference comparisons, muscle moment arms of the posterior deep masseter, medial and lateral pterygoid muscles showed errors of 1.556 mm, 1.756 mm, and 2.778 mm, respectively. On the left side, similar results were detected only for the medial (1.578 mm) and lateral (3.556 mm) pterygoid muscles.

No errors higher than 1.5 mm were detected for the method of moments variance estimator.

The intra- and interexaminer errors for the 3D mechanical advantage were generally less than 0.01 and 0.05, respectively.

Discussion

For the proposed 3D method to calculate the mechanical advantage for the masticatory system, the reliability and landmark coordinate errors, muscle and bite force vectors, muscle and load moment arms, and 3D mechanical advantage of seven masticatory muscles at two dental positions were calculated. Reliability related the magnitude of the measurement error for the observed measurements to the inherent variability in the ‘error free’, ‘true’, or underlying level of the quantity being measured [32]. Furthermore, the average mean of the absolute differences identified the continuous differences among the chosen measurements (systematic and random errors), and the method of moments variance estimator delivered an approximation of the random error. While systematic errors represent reproducible inaccuracies that lead to a measured value that is consistently larger or smaller than the true value, random errors lead to unpredictably variable differences [19]. Complementarily, the coefficient of variation quantifies an error variation relative to the mean and hence it can be used to compare the inconsistency of different magnitudes, and to establish the extent of differences expected in a certain trial due to measurement error [33].

Systematic and random errors were, however, partially controlled in the present study by calibration of the examiners and measuring instruments, and by averaging over a number of observations, respectively. If reliability was high, measurement errors were small in comparison to the true quantity being measured. Conversely, when the measurement errors tended to be large, reliability was low because differences between measurements are thought to be due purely to error rather than to a genuine difference in their true values [19, 30, 31, 33]. Reliability and errors for all variables in this study were determined with intra- and interexaminer comparisons from three examiners. As reliability depends on both the magnitude of measurement errors and the true heterogeneity in the population in which measurements are made, a homogeneous sample composed of healthy young people without severe craniofacial alterations was selected.

Landmarks showed excellent intra- and interexaminer reliability. Intraexaminer data exhibited errors < 1.5 mm, which is clinically acceptable [9]. Unsatisfactory interexaminer errors ranged from 1.51–5.83 mm. The use of 3 coordinates would allow for a more specific location; however, 3D images create additional difficulties due to the curvature of surfaces and no distinguishing limits between structures. In the 2D method, landmarks on curves can be resolved by bisectors from tangent lines and limits, which are determined by differences in radiopacity. The results for the identification of the landmarks in 3D are in accordance with previous studies, in which Orbitale, Porion, Gonion, Ramus, Pterygoid, and Pterygoid fovea landmarks were harder to recognize, showing higher errors [9, 21]. Better calibration and more experienced examiners to modify the bright, contrast and bone density of rendered images may be necessary to obtain better results.

The circular shape of the orbit for recognition of the Orbitale could explain the obtained errors due to the successive changes in image inclination and different perspectives during manipulation. Porion identification could generate confusion regarding the depth and the external and internal limits of the external acoustic meatus, although the degree of bone density can be altered. The end of the mandibular base and the beginning of the posterior border of the ascending ramus are tenuous during determination of Gonion and may worsen with image manipulation. Also, the width of the posterior border may identify the landmark on a more lateral or medial position [20]. The lack of clear limits on the concave surface between the ramus and body of the mandible may also have produced errors when localizing the Ramus. Moreover, the difficult definition of the lateral and medial pterygoid plates of the pterygoid process of

Table 5 Mean and standard deviation (SD), and intra- and interexaminer ratios between the mean and variability corresponding to biomechanical variables
Tab. 5 Mittelwert, Standardabweichung (SD) und Verhältnis zwischen Mittelwert und Variabilität der biomechanischen Variablen innerhalb und zwischen den Untersuchern

Biomechanical variables		Intraexaminer			Interexaminer		
		Mean	SD	CV (%)	Mean	SD	CV (%)
**Right side**							
Muscle moment arm (mm)	Superficial masseter	34.644	0.418	1.245	35.189	0.863	2.499
	Anterior deep masseter	32.622	0.322	1.041	32.722	0.690	2.112
	Posterior deep masseter	14.489	0.509	4.185	14.556	1.246	9.650
	Anterior temporal	32.489	0.322	1.052	32.567	0.767	2.341
	Posterior temporal	28.833	0.250	0.872	28.567	0.760	2.669
	Medial pterygoid	37.333	0.769	2.119	37.756	1.394	3.726
	Lateral pterygoid	10.156	0.649	6.533	10.556	2.201	**21.145**
Load moment arm (mm)	Molar	71.178	0.355	0.508	70.622	1.100	1.571
	Incisal	103.244	0.506	0.498	103.500	0.851	0.829
Mechanical advantage	Molar position						
	Superficial masseter	0.488	0.007	1.470	0.500	0.016	3.214
	Anterior deep masseter	0.459	0.005	1.129	0.464	0.011	2.295
	Posterior deep masseter	0.204	0.008	4.529	0.207	0.018	9.920
	Anterior temporal	0.457	0.005	1.098	0.462	0.012	2.517
	Posterior temporal	0.406	0.005	1.126	0.406	0.010	2.395
	Medial pterygoid	0.526	0.010	1.976	0.536	0.019	3.499
	Lateral pterygoid	0.144	0.009	6.542	0.150	0.031	**21.385**
	Incisal position						
	Superficial masseter	0.336	0.005	1.470	0.340	0.008	2.450
	Anterior deep masseter	0.316	0.003	1.060	0.316	0.007	2.069
	Posterior deep masseter	0.140	0.005	4.469	0.141	0.012	9.922
	Anterior temporal	0.315	0.004	1.152	0.315	0.007	2.195
	Posterior temporal	0.279	0.003	1.070	0.276	0.008	2.841
	Medial pterygoid	0.362	0.007	2.115	0.365	0.013	3.503
	Lateral pterygoid	0.099	0.006	6.480	0.102	0.021	**21.072**
**Left side**							
Muscle moment arm (mm)	Superficial masseter	34.778	0.606	1.755	35.333	1.018	2.975
	Anterior deep masseter	32.322	0.346	1.070	32.344	0.711	2.221
	Posterior deep masseter	14.244	0.507	3.581	14.000	1.179	8.291
	Anterior temporal	32.178	0.289	0.900	32.144	0.759	2.386
	Posterior temporal	28.311	0.289	1.052	28.100	0.804	2.899
	Medial pterygoid	37.756	0.485	1.308	37.944	1.268	3.367
	Lateral pterygoid	9.656	0.584	6.118	10.489	2.823	**27.849**
Load mechanical arm (mm)	Molar	71.256	0.555	0.794	70.611	1.050	1.497
	Incisal	103.089	0.578	0.559	103.300	1.097	1.066

Table 5 (Continued)
Tab. 5 (Fortsetzung)

Biomechanical variables		Intraexaminer			Interexaminer		
		Mean	SD	CV (%)	Mean	SD	CV (%)
Mechanical advantage	Molar position						
	Superficial masseter	0.489	0.010	2.157	0.501	0.018	3.540
	Anterior deep masseter	0.454	0.007	1.563	0.459	0.011	2.486
	Posterior deep masseter	0.201	0.008	3.763	0.199	0.018	8.780
	Anterior temporal	0.452	0.006	1.317	0.456	0.010	2.213
	Posterior temporal	0.398	0.006	1.417	0.399	0.010	2.584
	Medial pterygoid	0.531	0.008	1.578	0.538	0.017	3.078
	Lateral pterygoid	0.136	0.008	6.248	0.149	0.041	**28.694**
	Incisal position						
	Superficial masseter	0.338	0.006	1.932	0.342	0.010	2.994
	Anterior deep masseter	0.313	0.004	1.276	0.313	0.007	2.234
	Posterior deep masseter	0.138	0.005	3.790	0.136	0.012	8.666
	Anterior temporal	0.312	0.004	1.136	0.311	0.007	2.206
	Posterior temporal	0.275	0.003	1.227	0.272	0.007	2.783
	Medial pterygoid	0.366	0.005	1.342	0.367	0.011	3.072
	Lateral pterygoid	0.094	0.006	6.262	0.102	0.028	**28.172**

Coefficient of variation = 20–30% are shown in bold type
SD Standard deviation, *CV* Coefficient of variation

Table 6 Intra- and interexaminer reliability and errors to biomechanical variables
Tab. 6 Intra- und Inter-Untersucher-Reliabilität und Fehler bei biomechanischen Variablen

Biomechanical variables	Intraexaminer			Interexaminer		
	ICC*	AMAD	SDMAD	ICC*	AMAD	SDMAD
**Right side**						
Muscle moment arm (mm)						
Superficial masseter	0.991	0.489	0.389	0.968	1.067	0.734
Anterior deep masseter	0.994	0.378	0.379	0.975	0.844	0.630
Posterior deep masseter	0.988	0.600	0.405	0.843	**1.556**	1.447
Anterior temporal	0.994	0.378	0.379	0.972	0.933	0.596
Posterior temporal	0.994	0.289	0.336	0.968	0.933	0.621
Medial pterygoid	0.954	0.933	0.755	0.806	**1.756**	1.339
Lateral pterygoid	0.919	0.778	0.583	0.434†	**2.778**	1.466
Load moment arm (mm)						
Molar	0.997	0.422	0.446	0.982	1.378	0.820
Incisal	0.992	0.600	0.790	0.985	1.044	0.870
Mechanical advantage						
Molar position						
Superficial masseter	0.987	0.009	0.007	0.949	0.020	0.011
Anterior deep masseter	0.992	0.006	0.005	0.972	0.013	0.007
Posterior deep masseter	0.988	0.009	0.005	0.852	0.023	0.022
Anterior temporal	0.992	0.006	0.006	0.968	0.015	0.007
Posterior temporal	0.994	0.006	0.004	0.973	0.013	0.008
Medial pterygoid	0.957	0.012	0.011	0.836	0.023	0.021
Lateral pterygoid	0.943	0.011	0.008	0.394†	0.040	0.022
Incisal position						
Superficial masseter	0.986	0.006	0.004	0.958	0.010	0.008
Anterior deep masseter	0.992	0.004	0.003	0.967	0.008	0.005
Posterior deep masseter	0.987	0.006	0.004	0.834	0.016	0.015
Anterior temporal	0.990	0.004	0.003	0.960	0.009	0.006
Posterior temporal	0.991	0.004	0.003	0.951	0.010	0.006
Medial pterygoid	0.933	0.009	0.008	0.720†	0.016	0.013
Lateral pterygoid	0.934	0.008	0.005	0.397†	0.027	0.015

Table 6 (Continued)
Tab. 6 (Fortsetzung)

Biomechanical variables	Intraexaminer			Interexaminer		
	ICC*	AMAD	SDMAD	ICC*	AMAD	SDMAD
**Left side**						
Muscle moment arm (mm)						
Superficial masseter	0.967	0.733	0.750	0.921	1.289	1.174
Anterior deep masseter	0.993	0.400	0.332	0.978	0.867	0.558
Posterior deep masseter	0.966	0.600	0.663	0.852	1.467	1.027
Anterior temporal	0.993	0.333	0.382	0.973	0.933	0.513
Posterior temporal	0.992	0.333	0.339	0.966	0.956	0.545
Medial pterygoid	0.985	0.578	0.419	0.874	**1.578**	1.181
Lateral pterygoid	0.942	0.733	0.535	0.313†	**3.556**	1.709
Load moment arm (mm)						
Molar	0.992	0.667	0.554	0.975	1.289	0.741
Incisal	0.988	0.689	0.753	0.972	1.378	0.856
Mechanical advantage						
Molar position						
Superficial masseter	0.956	0.013	0.012	0.900	0.022	0.017
Anterior deep masseter	0.985	0.009	0.006	0.969	0.014	0.007
Posterior deep masseter	0.975	0.009	0.009	0.869	0.023	0.016
Anterior temporal	0.987	0.007	0.006	0.971	0.013	0.007
Posterior temporal	0.987	0.007	0.005	0.968	0.013	0.008
Medial pterygoid	0.973	0.011	0.006	0.870	0.021	0.019
Lateral pterygoid	0.953	0.011	0.007	0.341†	0.052	0.024
Incisal position						
Superficial masseter	0.959	0.008	0.007	0.915	0.013	0.011
Anterior deep masseter	0.986	0.005	0.004	0.968	0.009	0.005
Posterior deep masseter	0.969	0.006	0.006	0.860	0.015	0.010
Anterior temporal	0.985	0.004	0.004	0.963	0.009	0.005
Posterior temporal	0.985	0.004	0.004	0.958	0.009	0.005
Medial pterygoid	0.977	0.006	0.004	0.841	0.014	0.011
Lateral pterygoid	0.945	0.007	0.005	0.322†	0.035	0.016

Average values > 1.5 mm are shown in bold type
ICC Intraclass correlation coefficient, *AMAD* Average mean of absolute differences, *SDMAD* Standard deviation mean of absolute differences
*$p < 0.0001$, †$p < 0.01$

the sphenoid (concave and asymmetric surfaces) hampered Pterygoid determination at the midpoint of the lateral pterygoid plate. The concave surface and the convex ridges of the neck of condyle that gives rise to the mandibular notch and the beginning of the coronoid process converge simultaneously and also prevent a concise identification of the Pterygoid fovea. Because Maa and Map are derived from the localization of Gonion and Ramus, the positioning of these landmarks consequently affected their reliability and errors.

Biomechanical variables showed excellent and nearly excellent intraexaminer and interexaminer reliability, respectively, as well as a very low coefficients of variation. Although the muscle moment arm and the 3D mechanical advantage of the lateral pterygoid muscle had ICC values below 0.500, the intra- and interexaminer 3D mechanical advantage errors were ≤ 0.01 and ≤ 0.05, respectively. Likewise, the higher interexaminer variation for the lateral pterygoid muscle, even on the left side, may be considered acceptable for cephalometric clinical purposes. Reliability of the results for the muscle moment arms, and 3D mechanical advantages on the one hand depend on landmark localization, but the generally promising results can also be explained by a probable compensation resulting from the greater lengths of moment arms when compared to the magnitude of the error of landmark coordinates, which were smaller and imply greater precision. Thus, the obtained lower interexaminer ICC values for the left lateral pterygoid muscle could be explained by the resulting lower lengths of the muscle moment arms. The shorter length of this muscle possibly prevents the compensation described above. This is not the case with the longer muscle moment arms from the other variables, which presented lower coefficients of variation than in studies using lateral radiographs [4, 5, 34] or magnetic resonance images [6, 10, 11, 28].

The orientation and sense of the muscle force vectors can also affect the 3D mechanical advantage calculation [2–6, 8]. The whole muscles were represented in the present study by the average fiber vectors attached to the anatomical landmarks. It is necessary to interpret this method as a reasonable simplification because the muscular action lines do not completely correspond to a single muscular isometric work [26], especially in muscles with a complex internal organization [35]. Likewise, a class III lever cannot describe all mandibular movements, which are much more complex than a hinge pattern. However, it would almost be impossible to analyze all biomechanical variables and mandibular positions before and after treatment that could eventually alter the patients' mandibular mechanical advantage. Deeper analysis, perhaps not as part of daily clinical practice, could be performed in biomechanical research. On the other hand, the present method is intended to be applied clinically, but with sufficient physiological plausibility and scientific rigor.

Changes in mechanical advantage according to different craniofacial characteristics can be described using a 2D method. It is known that an upward position of the maxilla (hypodivergent growth pattern) will tend to increase the mechanical advantage of the elevator muscles, while a downward position (hyperdivergent growth pattern) will have the opposite effect [2, 13]. Advancement of the maxilla will decrease the mechanical advantage of the elevator muscles by changing the relationship of the bite position to the condyles and increasing the load moment arms [2]. A reduced mechanical advantage could also be observed in long face subjects (mandibular plane $\geq 40°$), who sometimes require bimaxillary surgery to induce counterclockwise rotation of the mandible [4–6]. However, results from magnetic resonance images indicated that directional changes of the muscle moment arms and a decrease of the 3D mechanical advantage of the masseter and medial pterygoid muscle occurred after surgical advancement and posterior intrusion of mandible with anterior rotation of the proximal segment [10, 11, 28].

The intention of the present study was to verify the methodological feasibility of the recommended method [36], but not to determine epidemiological data of a certain population. Thus, variables were barely compared one to another. The fact that one examiner trained all the other examiners might be thought about to have induced possible bias. Perhaps the second and third examiners could have adopted the "style" of main examiner. However, the intention of this study was to evaluate the differences of reproducibility and errors (by biases) among an expert and other professionals, showing the versality of the method for different applications. A bias would have been manifest if this study had the objective to compare the means of mechanical advantage measurements between groups with contrasting features. In this case, a measurement bias can exist. The examiners were professors belonging to the same educational institution, inevitably having the same philosophy in several procedures.

The next step for this method will be to determine its validity, comparing the average mean of absolute differences of 3D values with conventional cephalometric data. Also, sensitivity and specificity of the method in patients with malocclusions and facial deformities will have to be evaluated to determine its application profile. The equivalence of the mechanical advantage with bite force could also be calculated, establishing the influence of moment arms on function [34, 37].

The present method is a new proposal and it was only described previously for radiographs or MRIs. Only one study using CBCT assumed that certain types of malocclusion may have different masseter lengths and orientations

and that these differences may have implications for the mechanical advantage of bite force [13]. The method may be of interest to biologists and functional morphologists, since it can potentially describe biomechanical changes during craniofacial growth and development [38]. However, it should also be interesting for clinicians who seek to intervene in dysfunctional situations [18]. Orthodontists, orthopedists, and surgeons may considerer analyzing the changes in the 3D mechanical advantage as a complementary study for diagnosis and treatment planning of facial disharmonies [2, 3], as well as to identify possible normalization of the mandibular mechanical advantage after improvement of the skeletal relationship [4–6, 8, 10, 11].

Conclusions

Measurement of the mechanical advantage applying the proposed cone-beam computed tomography images-based method showed an overall high intra- and interexaminer reliability and acceptable errors.

Supplementary Information The online version of this article (https://doi.org/10.1007/s00056-022-00378-7) contains supplementary information, which is available to authorized users.

Declarations

Conflict of interest A. Sánchez-Ayala, A. Sánchez-Ayala, R.C. Kolodzejezyk, V.M. Urban, M.Ó. Lagravère and N.H. Campanha declare that they have no competing interests.

Ethical standards All procedures performed in studies involving human participants or on human tissue were in accordance with the ethical standards of the institutional and/or national research committee and with the 1975 Helsinki declaration and its later amendments or comparable ethical standards. This work was approved by the Health Research Ethics Board at the University of Alberta (#5563). All subjects who participated in the study signed written informed consent to participate.

References

1. Hamill J, Knutzen KM, Derrick TR (2014) Biomechanical basis of human movement, 4th edn. Wolters Kluwer Health Adis, Philadelphia
2. Throckmorton GS, Finn RA, Bell WH (1980) Biomechanics of differences in lower facial height. Am J Orthod 77:410–420. https://doi.org/10.1016/0002-9416(80)90106-2
3. Throckmorton GS, Dean JS (1994) The relationship between jaw-muscle mechanical advantage and activity levels during isometric bites in humans. Arch Oral Biol 39:429–437. https://doi.org/10.1016/0003-9969(94)90174-0
4. García-Morales P, Buschang PH, Throckmorton GS, English JD (2003) Maximum bite force, muscle efficiency and mechanical advantage in children with vertical growth patterns. Eur J Orthod 25:265–272. https://doi.org/10.1093/ejo/25.3.265
5. Charalampidou M, Kjellberg H, Georgiakaki I, Kiliaridis S (2008) Masseter muscle thickness and mechanical advantage in relation to vertical craniofacial morphology in children. Acta Odontol Scand 66:23–30. https://doi.org/10.1080/00016350701884604
6. van Spronsen PH (2010) Long-face craniofacial morphology: cause or effect of weak masticatory musculature? Semin Orthod 16:99–117. https://doi.org/10.1053/j.sodo.2010.02.001
7. Sagl B, Schmid-Schwap M, Piehslinger E et al (2019) A dynamic jaw model with a finite-element temporomandibular joint. Front Physiol 10:1–12. https://doi.org/10.3389/fphys.2019.01156
8. Thomas GP, Throckmorton GS, Ellis E, Sinn DP (1995) The effects of orthodontic treatment on isometric bite forces and mandibular motion in patients before orthognathic surgery. J Oral Maxillofac Surg 53:673–678. https://doi.org/10.1016/0278-2391(95)90168-X
9. Lagravère MO, Gordon JM, Guedes IH et al (2009) Reliability of traditional cephalometric landmarks as seen in three-dimensional analysis in maxillary expansion treatments. Angle Orthod 79:1047–1056. https://doi.org/10.2319/010509-10R.1
10. Dicker GJ, Koolstra JH, Castelijns JA et al (2012) Positional changes of the masseter and medial pterygoid muscles after surgical mandibular advancement procedures: An MRI study. Int J Oral Maxillofac Surg 41:922–929. https://doi.org/10.1016/j.ijom.2012.01.007
11. Dicker GJ, Tuijt M, Koolstra JH et al (2012) Static and dynamic loading of mandibular condyles and their positional changes after bilateral sagittal split advancement osteotomies. Int J Oral Maxillofac Surg 41:1131–1136. https://doi.org/10.1016/j.ijom.2012.03.013
12. O'Neil R, Kau CH (2021) Comparison of dental arch forms created from assessment of teeth, alveolar bone, and the overlying soft tissue. J Orofac Orthop. https://doi.org/10.1007/s00056-021-00282-6
13. Becht MP, Mah J, Martin C et al (2014) Évaluation De La Morphologie Du Muscle Masséter Dans Différents Types De Malocclusions En Utilisant La Tomographie Volumétrique À Faisceau Conique. Int Orthod 12:32–48. https://doi.org/10.1016/j.ortho.2013.12.003
14. Velásquez RL, Coro JC, Londoño A et al (2018) Three-dimensional morphological characterization of malocclusions with mandibular lateral displacement using cone-beam computed tomography. Cranio 36:143–155. https://doi.org/10.1080/08869634.2017.1300994
15. Luebbert J, Ghoneima A, Lagravère MO (2016) Skeletal and dental effects of rapid maxillary expansion assessed through three-dimensional imaging: a multicenter study. Int Orthod 14:15–31. https://doi.org/10.1016/j.ortho.2015.12.013
16. Draenert FG, Erbe C, Zenglein V et al (2010) 3D analysis of condylar position after sagittal split osteotomy of the mandible in mono- and bimaxillary orthognathic surgery—a methodology study in 18 patients. J Orofac Orthop 71:421–429. https://doi.org/10.1007/s00056-010-1021-9
17. Alhammadi MS, Elfeky HY, Fayed MS et al (2019) Three-dimensional skeletal and pharyngeal airway changes following therapy with functional appliances in growing skeletal Class II malocclusion patients. J Orofac Orthop 80:254–265. https://doi.org/10.1007/s00056-019-00185-7
18. Haskell B, Day M, Tetz J (1986) Computer-aided modeling in the assessment of the biomechanical determinants of diverse skeletal patterns. Am J Orthod 89:363–382. https://doi.org/10.1016/0002-9416(86)90068-0
19. Springate SD (2012) The effect of sample size and bias on the reliability of estimates of error: a comparative study of Dahlberg's formula. Eur J Orthod 34:158–163. https://doi.org/10.1093/ejo/cjr010
20. Lagravère MO, Low C, Flores-Mir C et al (2010) Intraexaminer and interexaminer reliabilities of landmark identification on digitized lateral cephalograms and formatted 3-dimensional cone-beam

computerized tomography images. Am J Orthod Dentofac Orthop 137:598–604. https://doi.org/10.1016/j.ajodo.2008.07.018
21. Naji P, Alsufyani NA, Lagravère MO (2014) Reliability of anatomic structures as landmarks in three-dimensional cephalometric analysis using CBCT. Angle Orthod 84:762–772. https://doi.org/10.2319/090413-652.1
22. Zhang X, Ye L, Li H et al (2018) Surgical navigation improves reductions accuracy of unilateral complicated zygomaticomaxillary complex fractures: a randomized controlled trial. Sci Rep 8:1–9. https://doi.org/10.1038/s41598-018-25053-z
23. Coombs MC, Bonthius DJ, Nie X et al (2019) Effect of measurement technique on TMJ mandibular condyle and articular disc morphometry: CBCT, MRI, and physical measurements. J Oral Maxillofac Surg 77:42–53. https://doi.org/10.1016/j.joms.2018.06.175
24. Lagravère MO, Major PW (2005) Proposed reference point for 3-dimensional cephalometric analysis with cone-beam computerized tomography. Am J Orthod Dentofac Orthop 128:657–660. https://doi.org/10.1016/j.ajodo.2005.07.003
25. Baron P, Debussy T (1979) A biomechanical functional analysis of the masticatory muscles in man. Arch Oral Biol 24:547–553. https://doi.org/10.1016/0003-9969(79)90134-1
26. Koolstra JH, van Eijden TMGJ, van Spronsen PH et al (1990) Computer-assisted estimation of lines of action of human masticatory muscles reconstructed in vivo by means of magnetic resonance imaging of parallel sections. Arch Oral Biol 35:549–556. https://doi.org/10.1016/0003-9969(90)90086-P
27. Van Eijden TMGJ, Korfage JAM, Brugman P (1997) Architecture of the human jaw-closing and jaw-opening muscles. Anat Rec 248:464–474
28. Tuijt M, Koolstra JH, Lobbezoo F, Naeije M (2010) Differences in loading of the temporomandibular joint during opening and closing of the jaw. J Biomech 43:1048–1054. https://doi.org/10.1016/j.jbiomech.2009.12.013
29. Nicolielo LFP, Van Dessel J, Shaheen E et al (2017) Validation of a novel imaging approach using multi-slice CT and cone-beam CT to follow-up on condylar remodeling after bimaxillary surgery. Int J Oral Sci 9:139–144. https://doi.org/10.1038/ijos.2017.22
30. Kim H-Y (2013) Statistical notes for clinical researchers: Evaluation of measurement error 1: using intraclass correlation coefficients. Restor Dent Endod 38:98. https://doi.org/10.5395/rde.2013.38.2.98
31. Maspero C, Abate A, Bellincioni F et al (2019) Comparison of a tridimensional cephalometric analysis performed on 3T-MRI compared with CBCT: a pilot study in adults. Prog Orthod 20:40. https://doi.org/10.1186/s40510-019-0293-x
32. Bartlett JW, Frost C (2008) Reliability, repeatability and reproducibility: Analysis of measurement errors in continuous variables. Ultrasound Obstet Gynecol 31:466–475. https://doi.org/10.1002/uog.5256
33. Shechtman O (2013th) The coefficient of variation as an index of measurement reliability. In: Doi SAR, Williams GM (eds) Methods of clinical epidemiology. Springer, Heidelberg, New York, pp 39–49 https://doi.org/10.1007/978-3-642-37131-8_4
34. Throckmorton GS, Ellis E (2001) The relationship between surgical changes in dentofacial morphology and changes in maximum bite force. J Oral Maxillofac Surg 59:620–627. https://doi.org/10.1053/joms.2001.23373
35. Hannam AG, McMillan AS (1994) Internal organization in the human jaw muscles. Crit Rev Oral Biol Med 5:55–89. https://doi.org/10.1177/10454411940050010301
36. Castro IO, Gribel FB, de Alencar AHG et al (2018) Evaluation of crown inclination and angulation after orthodontic treatment using digital models. J Orofac Orthop 79:227–234. https://doi.org/10.1007/s00056-018-0136-2
37. Takeuchi-Sato T, Arima T, Mew M, Svensson P (2019) Relationships between craniofacial morphology and masticatory muscle activity during isometric contraction at different interocclusal distances. Arch Oral Biol 98:52–60. https://doi.org/10.1016/j.archoralbio.2018.10.030
38. Du W, Bhojwani A, Hu JK (2021) FACEts of mechanical regulation in the morphogenesis of craniofacial structures. Int J Oral Sci 13:1–16. https://doi.org/10.1038/s41368-020-00110-4

Publisher's Note Springer Nature remains neutral with regard to jurisdictional claims in published maps and institutional affiliations.
